# Antiplasmodial Properties of Aqueous and Ethanolic Extracts of Ten Herbal Traditional Recipes Used in Thailand against *Plasmodium falciparum*

**DOI:** 10.3390/tropicalmed7120417

**Published:** 2022-12-04

**Authors:** Arisara Phuwajaroanpong, Prapaporn Chaniad, Walaiporn Plirat, Sathianpong Phoopha, Abdi Wira Septama, Arnon Chukaew, Chuchard Punsawad

**Affiliations:** 1 Department of Medical Sciences, School of Medicine, Walailak University, Nakhon Si Thammarat 80160, Thailand; 2 Research Center in Tropical Pathobiology, Walailak University, Nakhon Si Thammarat 80160, Thailand; 3 Traditional Thai Medical Research and Innovation Center, Faculty of Traditional Thai Medicine, Prince of Songkla University, Songkhla 90110, Thailand; 4 Center for Pharmaceutical Ingredient and Traditional Medicine, National Research and Innovation Agency (BRIN), Cibinong Science Center, Bogor 16915, Indonesia; 5 Chemistry Department, Faculty of Science and Technology, Suratthani Rajabhat University, Surat Tani 84100, Thailand

**Keywords:** herbal recipe, antimalarial activity, antiplasmodial activity, cytotoxicity, hemolysis, oxidant

## Abstract

This study evaluated the in vitro and in vivo antiplasmodial efficacy and toxicity of aqueous and ethanolic extracts from traditional recipes used in Thailand. The aqueous and ethanolic extracts of ten traditional recipes were tested for in vitro antiplasmodial activity (parasite lactate dehydrogenase assay), cytotoxicity (MTT assay), and hemolysis). Oxidant levels were measured using cell-permeable probe 5-(and-6)-chloromethyl-2′,7′-dichlorodihydrofluorescein diacetate fluorescent dye-based assays. The best candidate was chosen for testing in mouse models using 4-day suppressive and acute toxicity assays. An in vitro study showed that ethanolic extracts and three aqueous extracts exhibited antiplasmodial activity, with an IC_50_ in the range of 2.8–15.5 µg/mL. All extracts showed high CC_50_ values, except for ethanolic extracts from Benjakul, Benjalotiga, and Trikatuk in HepG2 and Benjalotiga and aqueous extract from Chan-tang-ha in a Vero cell. Based on the results of the in vitro antiplasmodial activity, an aqueous extract of Triphala was chosen for testing in mouse models. The aqueous extract of Triphala exhibited good antiplasmodial activity, was safe at an oral dose of 2 g/kg, and is a potential candidate as a new source for the development of antimalarial drugs.

## 1. Introduction

The malaria burden has an impact around the world and is the heaviest, according to the World Health Organization (WHO). The African region accounted for approximately 95% of all cases and 96% of all deaths in 2020 [1]. There are five *Plasmodium* species that cause devastation to human life—*P. falciparum*, *P. vivax*, *P. malariae*, *P. ovale*, and *P. knowlesi*. A key feature of these infections is their ability to invade erythrocytes [2]. Sequestered parasites can lead to impaired blood flow and induce negative effects on critical organs [3]. However, effective tools for malaria control and elimination rely on potent antimalarial agents. The WHO recommends six artemisinin combinations (ACTs) as first-line treatment for uncomplicated falciparum malaria: artemether-lumefantrine, artesunate-amodiaquine, artesunate-mefloquine, artesunate-sulfadoxine-pyrimethamine, dihydroartemisinin-piperaquine, and artesunate-pyronaridine [4]. Blood-stage malaria plays a crucial role in the development of symptoms and clinical complications and can be terminated by blood schizonticides. Unfortunately, artemisinin and partner drugs slow parasite clearance along the Thai-Cambodian and Thai-Myanmar borders and spread to other areas [5]. The emergence of resistance makes malaria control and elimination challenging, highlighting the need for novel drug strategies. Traditional herbal medicine comprises pharmaceutical agents and is still commonly used to reduce the risk of disease in rural areas around the world [6]. The reasons for the use of herbal medicines are their safety, effectiveness, cultural preferences, inexpensiveness, and easy availability [7]. Today, traditional healers are still active, play a role in healing health in Thai society, and can be found in all parts of Thailand [6,8]. The concept of traditional therapeutic herbal strategies is based on two principles that involve single and polyherbal (herbal recipe) methods [9]. Even though individual herbs confer some benefits, herbal recipes evidently provide extra therapeutic effects from positive synergistic interaction [9].

In Ayurveda literature, one of the traditional medicinal systems in India, plant combinations are chosen rather than individual plants because of their ability to produce a greater result of varying potency [9]. Triphala and Trikatuk are traditional herbal recipes widely used in Ayurvedic and Thai traditional medicine. Triphala is commercially available and is recommended to treat several health ailments, such as fever, cough, asthma, jaundice, anemia, inflammation, cardiovascular disorders, and liver dysfunction [10,11]. Trikatuk has been used to treat a wide range of illnesses owing to its anti-allergic, anti-inflammatory, anticholinesterase, and antioxidant effects [12,13]. Trisamo has analgesic, antipyretic, antibacterial, and antioxidant properties; promotes general health; and is used as an antipyretic in Thai traditional medicine [14]. Jatu-phala-tiga possesses strong free-radical-scavenging properties [15]. Benjakul is a Thai herbal recipe on the Thailand National List of Essential Medicines. This recipe possesses anti-allergic, anti-inflammatory, and anticancer activities [16]. In Thai traditional medicine, Benjalotiga, Gaysorn-tang-ha, Benjathian, Benjagot, and Chan-tang-ha recipes are widely used in primary health care and associated with potential health benefits such as antipyretic, cardio-tonic, and hematic tonic prescriptions.

Interestingly, the multiple health-promoting properties of herbal medicines have inspired us to discover the elements as key antimalarial agents, and ten existing traditional medicines used in Thailand have not yet been investigated for their antimalarial activities. Therefore, the aim of this study was to evaluate the antiplasmodial activities of ten traditional recipes against *P. falciparum* infection in in vitro cultures and assess the antimalarial activity and acute toxicity of a good candidate in mouse models.

## 2. Materials and Methods

### 2.1. Herbal Material and Traditional Recipe

The 32 plants shown in Table 1 were authorized by a botanist after being obtained from a traditional Thai drug store in Muang District, Nakhon Si Thammarat Province, southern Thailand. The plant identification was performed using morphological characteristics and also confirmed by comparison with the herbarium specimens. The authorization for plant materials complied with the relevant guidelines and regulations of the Plant Varieties Protection, Department of Agriculture, Ministry of Agriculture and Cooperatives, Thailand. Voucher specimens were identified and deposited at the Department of Medical Sciences, School of Medicine, Walailak University, Thailand. The plants were washed with tap water and then dried in a hot air oven (Memmert, Model; SFE600, Schwabach, Germany). The plant parts were ground into a fine powder using a grinder (Taizhou Jincheng Pharmaceutical Machinery Co., Ltd., Model; SF, Jiangsu, China). The particle size of the powdered plant was 2.36 mm. Ten traditional herbal recipes were prepared by blending equal portions of the ingredients [17,18]. The final weight was made at 60 g to achieve an adequate quantity of crude extract.

### 2.2. Preparation of Crude Extract

The extraction process was performed as previously described [19]. The maceration method was performed to prepare ethanolic extracts. Sixty grams of the powdered recipe was extracted for 72 h in 600 mL of 80% ethanol at 25 °C. The decoction method was used to make aqueous extracts. Sixty grams of each herb powder was extracted three times by mixing with 600 mL of distilled water and allowed to boil for 30 min. Subsequently, the liquid portion was separated from the residue using filter paper (Whatman, Buckinghamshire, England). The marcs of ethanolic and aqueous extract were re-extracted twice, with 600 mL of solvents each time. The filtrates were combined and concentrated using a rotary evaporator (Rotavapor, Buchi, China) at 45 rpm and 45 °C. Further drying was performed in a freeze-drying machine at −89 °C (Martin Christ, Germany). The crude extracts were stored in a refrigerator until further use.

### 2.3. Phytochemical Analysis

Twenty different extracts were tested for phytochemicals according to standard procedures, as previously described [20,21]. Qualitative phytochemical screening was performed for flavonoids, terpenoids, alkaloids, tannins, anthraquinone, cardiac glycosides, saponins, and coumarins.

### 2.4. In Vitro Culture of Plasmodium Parasites

K1 chloroquine-resistant *P. falciparum* strain was used in this study. The cryopreserved parasite-infected blood was thawed using a 12% and 1.6% NaCl concentration gradient. The culture of the parasite was slightly modified from the original methods developed by Trager and Jensen [22]. It was cultured in a T-75 flask containing 2% human O+ erythrocytes, base media of RPMI-1640 medium (Gibco, Carlsbad, CA, USA) supplemented with 2 mg/mL NaHCO_3_, 4.8 mg/mL HEPES (Himedia, Mumbai, India), 10 μg/mL hypoxanthine (Sigma-Aldrich, New Delhi, India), 2.5 μg/mL gentamicin (Sigma-Aldrich, New Delhi, India), and 0.5% albumax II (Gibco MA, USA) in a saturated atmosphere of 5% CO_2_ at 37 °C. In order to assess parasitemia and developmental stages, a thin blood smear was stained with Giemsa dye, and intracellular parasites were visualized under an oil immersion lens (100×) using a light microscope (Olympus CX31, Model CX31RBSFA, Tokyo, Japan).

### 2.5. Cell Culture and Maintenance

The HepG2 cells obtained from the ATCC cell bank (HB-8065™) and Vero cells (Elabscience, Wuhan, Hubei, China) were individually cultured in DMEM media (Gibco, Carlsbad, CA, USA) supplemented with 10% FBS (Sigma-Aldrich, New Delhi, India), 1% (*v*/*v*) penicillin/streptomycin (Sigma-Aldrich, St Louis, MO, USA) at 37 °C in an atmosphere of 5% CO_2_. The cell confluence and morphology were observed using a phase-contrast inverted microscope (Olympus, Model CK X31, Hicksville, NY, USA). Once the cells reached approximately 80% confluence, 2.5% trypsin-EDTA (Gibco, Carlsbad, CA, USA) was used to detach cells before subculturing.

### 2.6. Detection of Parasite Lactate Dehydrogenase (pLDH) Activity

A stock solution (20 mg/mL) of the aqueous extracts was prepared by dissolving the extract in phosphate-buffered saline (PBS), whereas the ethanolic extracts were dissolved in DMSO (dimethyl sulfoxide). A two-fold serial dilution was prepared using PBS (aqueous extracts) or DMSO (ethanolic extracts), covering a range of final concentration from 1.56 to 100 µg/mL. The antiplasmodial activity was determined by the concentration of the extracts that inhibited 50 percent of the parasite growth (IC_50_) by the measurement of pLDH activity, as previously described [23]. The crude extract (1µL) was incubated with 1% parasitized red blood cells (pRBCs) and 2% hematocrit (199 µL) in a 96-well cell culture plate (SPL Life Sciences, Pocheon-si, Gyeonggi-do, Korea) in an atmosphere of 5% CO_2_ at 37 °C for 72 h. The test was performed in triplicate for each concentration. Artesunate (Sigma, St Louis, MO USA) was used as the positive control (final concentration ranging from 1.56 to 100 ng/mL). PBS and DMSO were used as negative controls. Non-infected red blood cells served as blank controls. The suspension was frozen at −80 °C and thawed at 37 °C to lyse red cell pellets. The pLDH enzyme was detected by the reaction between 20 µL of their contents from the released red cells, 100 µL of malstate reagent, and 20 µL of nitroblue tetrazolium/phenazine ethosulfate solution (Calbiochem, Sigma-Aldrich, New Delhi, India) in a new 96-well plate flat bottom. The absorbance was measured at 650 nm (Biotek Eon, Winooski, VT, USA) after the reaction was incubated in the dark for an hour. The percent inhibition was calculated compared to the negative control after subtraction of the background using the following formula:

% inhibition = 100 × [(OD negative well − OD sample well)/OD negative well]

IC_50_ was determined for each sample by plotting % growth as a function of concentration and estimating the concentration which caused 50% growth inhibition.

### 2.7. Cytotoxicity Assessment by 3-[4,5-Dimethylthiazol-2-yl]-2,5 diphenyl Tetrazolium Bromide (MTT) Assay

Crude extracts were evaluated for toxicity at concentrations ranging from 12.5 to 800 µg/mL. Serial dilutions were prepared using a 160 mg/mL stock solution. Cell cultures at a density of 10^4^ cells/well in 200 μL culture medium were seeded in a 96-well cell culture plate and then incubated at 37 °C for 24 h in an atmosphere of 5% CO_2_ until they reached confluence. HepG2 and Vero cell lines (199 µL) were treated with different concentrations of the extract (1 µL), with doxorubicin (final concentration ranging from 0.3 to 20 µg/mL) (Sigma-Aldrich, New Delhi, India) as a positive control. DMSO and PBS served as negative controls for ethanolic and aqueous extracts. Treated cells were incubated in an incubator at 37 °C for 48 h. The assays were performed in triplicates. MTT reagent (5 mg/mL) was added to each well at the end of the exposure period and then incubated at 37 °C for 3 h. Subsequently, the reagent was removed and replaced with 100 µL of DMSO. The absorbance was read at a wavelength of 590 nm using a microplate reader. The percent cytotoxicity was calculated to determine the cytotoxicity as follows:

% cytotoxicity = 100 − [100 × (OD sample well/OD negative well)]

The data in this assay are presented as the 50% cytotoxicity concentration (CC_50_), as determined by regression analysis using GraphPad Prism 6.

### 2.8. Hemolysis Measurement

The toxicity of crude extracts in human erythrocytes was evaluated by monitoring the hemolysis of the red cell suspension. Venous blood was drawn from healthy donors into EDTA-blood collection tubes, and the red cell pellet was washed three times with PBS. The plasma and buffy coat were discarded after centrifugation at 3000 rpm for 5 min. Cell suspension at 2% hematocrit was incubated with 50 µg/mL of the extracts in a final volume of 0.2 mL in an incubator at 37 °C for 72 h. Triton X-100 (Sigma-Aldrich, New Delhi, India) was used as a positive control. DMSO and PBS were used as negative controls. The plate was centrifuged at 3000 rpm for 5 min, and the supernatant was transferred to a new 96-well plate. The release of hemoglobin was measured at 570 nm, and the percentage of hemolysis was calculated as follows:

% hemolysis = [100 × (OD sample well − OD negative well)/(OD positive well − OD negative well)]

### 2.9. Measurement of Intracellular Oxidant in pRBCs

Oxidant generation was measured using the cell-permeable probe 5-(and-6)-chloromethyl-2′,7′-dichlorodihydrofluorescein diacetate (CM-H2DCFDA) (Invitrogen, Carlsbad, CA, USA). In this study, parasite culture at 1% parasitemia and 2% hematocrit was incubated with the presence of proximity IC_50_ concentration of drug or crude extracts in a 96-well plate for 72 h. Artesunate was used as the positive control. Negative controls were obtained using 0.5% DMSO and PBS. pRBCs were labeled with a fluorescence probe after disposal of the medium. The pellet was incubated in the dark with 100 µL CM-H2DCFDA solution at a final concentration of 10 mM for 30 min. Labeled cells were imaged using a Leica TCS SP5 confocal microscope (Leica, Mannheim, Germany) at an excitation/emission wavelength of 488/520 nm. In order to measure oxidant levels, five fields of each sample were acquired, and two hundred and twenty-five cells per sample were used for analysis. The integrated density, cell area, and background fluorescence were measured using the free software ImageJ Fiji. Corrected total cell fluorescence (CTCF), in terms of normalized values, was calculated as follows:

CTCF = integrated density − (area of selected cell × mean fluorescence of background readings).

The relative change was obtained by the division of CTCF from negative controls.

### 2.10. In Vivo Antimalarial Activity Model

The antimalarial efficacy of the crude extract against early malarial infection was evaluated using Peters’ 4-day suppressive test [24]. Male ICR mice (*n* = 25) aged 6–8 weeks were purchased from Nomura Siam International Co., Ltd., Bangkok, Thailand. They were randomly divided into five groups of five mice each. Group I was treated with PBS. Group II was treated with 25 mg/kg of chloroquine as the standard drug. Groups III, IV, and V received the aqueous extract of Triphala at doses of 200, 400, and 600 mg/kg, respectively. The animals were acclimatized for a week in the presence of food pellets and clean drinking water ad libitum before initiating the experiments. The *Plasmodium berghei* (*P. berghei*) ANKA strain (chloroquine-sensitive) was contributed by Thomas F. McCutchan and obtained from BEI Resources, NIAID, NIH. Mice infected with *P. berghei* were used as a donor, and then 25 mice were injected with 0.2 mL of 10^7^ infected red cells per milliliter through the intraperitoneal route. Oral administration started at 3 h post-infection, followed by 24, 48, and 72 h daily. On day 4, blood samples from each mouse were collected to prepare a thin blood film and stained with Giemsa solution. The percentages of parasitemia and suppression were calculated using the following formulas:

% parasitemia = (number of infected red blood cells/number of total red blood cells) × 100

% suppression = [(mean parasitemia_negative control_ − mean parasitemia_experimental group_)/mean parasitemia_negative control_] × 100

### 2.11. Acute Toxicity Test

The test was conducted according to the Organization for Economic Co-operation and Development (OECD) guidelines for testing chemicals, with a limit test at a dose of 2 g/kg (1). Ten male ICR mice were randomly divided into two groups: PBS solution and extract solution. A single oral dose was administered directly to the stomach through a feeding tube. Abnormalities were observed within the first 30 min and once daily for 14 days after administration. The parameters that were observed for signs of toxicity included general activity, body weight changes, tremors, convulsions, ataxia, diarrhea, urination, changes in skin fur, and death. On the last day, mice were anesthetized with 2% isoflurane and then euthanized by cardiac puncture after opening the thorax cavity. Blood samples were collected and placed into serum clot activator tubes for the analysis of liver and kidney enzymes, including aspartate aminotransferase (AST), alanine aminotransferase (ALT), alkaline phosphatase (ALP), blood urea nitrogen (BUN), and creatinine. The analysis was performed using an AU480 chemistry analyzer (Beckman Coulter, USA). In addition, histological alterations of the liver and kidneys were performed using hematoxylin and eosin staining as previously described [25]. The relative weights of the liver and kidneys were calculated using the following formula:

Relative organ weight = (organ weight/body weight) × 100

### 2.12. The Liquid Chromatography-Quadrupole Time-of-Flight Mass Spectrometry (LC-QTOF-MS) Analysis

Product profiling of the crude extract was performed using an LC-QTOF-MS instrument (1290 Infinity II LC-6545 Quadrupole-TOF, Agilent Technologies, Santa Clara, CA, USA). Chromatographic separation was performed on a Zorbax Rapid Resolution HD Eclipse Plus C18 column (150 mm length × 2.1 mm inner-diameter, particle size 1.8 μm) from Agilent (Agilent, Waldbronn, Germany). The elution gradient was performed with 0.1% formic acid water (mobile phase A) and acetonitrile (mobile phase B) at a flow rate of 0.20 mL/min. The column was equilibrated (A: B; v/v) at 90:10 (1 min), and elution was carried out with the following steps; 80:20 (from 1 to 12 min), 75:25 (from 12 to 20 min), 70:30 (from 20 to 25 min), 65:35 (from 25 to 28 min), 60:40 (from 28 to 38 min), and 90:10 (from 38 to 45 min). The column temperature was set to 25 °C. The instrument was set to an MS range of 100–1200 m/z in both negative and positive modes. The injection volume was 2 μL. Data acquisition was controlled using the MassHunter WorkStation Qualitative Analysis Workflows V8 software (Agilent Technologies, Santa Clara, CA, USA). Compounds were identified by comparing retention times, mass data, and fragmentation patterns with a compound database in the library search of the Mass Hunter METLIN database (Agilent Technologies). The peak with similarity scores of 90% compared to the database was selected to confirm peak identification.

### 2.13. Statistical Analysis

The data are expressed as the mean ± standard error of the mean. The relative CTCF was tested for normality before statistically assessing differences with an independent *t*-test; *p* < 0.05 was considered significant using SPSS for Microsoft Windows (version 17.0; IBM, Armonk, NY, USA). Statistical analysis of in vivo studies was performed using one-way analysis of variance at *p* < 0.05 after the values showed a normal distribution using SPSS for Microsoft Windows version 17.0.

## 3. Results

### 3.1. Percentage Yield of Crude Plant Material

The percentage yield of each extract obtained from the dried crude extract divided by the initial amount of powdered plant part is presented in Table 2. The maximum extract yield (40.12%) was obtained from the aqueous extract of the Jatu-phala-tiga recipe, whereas the minimum yield (2.07%) was recorded for the ethanolic extract of the Benjatian recipe. The yield of aqueous extracts from Benjalotiga and Chan-tang-ha was lower than that of the ethanolic extract; however, other extracts exhibited a higher percentage yield than that of the ethanolic extract.

### 3.2. Phytochemical Profile

**Figure 1:**
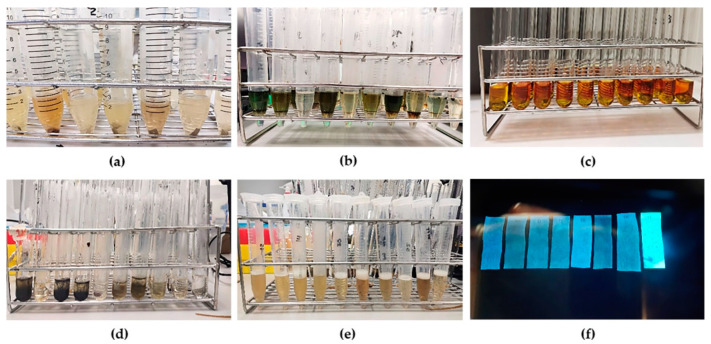
Qualitative phytochemical screening (**a**) screening for flavonoid, (**b**) screening for terpenoid, (**c**) screening for alkaloid, (**d**) screening for tannin, (**e**) screening for saponin, (**f**) screening for coumarin.

Qualitative analysis of the ten traditional recipes (aqueous/ethanolic extracts) revealed different phytocomponents, which are displayed in Table 3 and Figure 1. All extracts, except the ethanolic extracts from Trikatuk, Benjalotiga, Benjakul, and Chan-tang-ha, contained tannins. A majority of terpenoids were found in all extracts, excluding the aqueous extract from Benjagot. Anthraquinones and cardiac glycosides were not detected. Saponins were mainly found in the aqueous extracts.

### 3.3. In Vitro Antiplasmodial Activity

The antiplasmodial activities of the ten traditional recipes are shown in Table 4. According to these criteria of in vitro antiplasmodial assessment of products derived from plants [26], extracts with IC_50_ < 5 µg/mL are considered as exhibiting potent antiplasmodial activity, IC_50_ between 5–15 µg/mL and 15–50 µg/mL as good and moderate activity, respectively, and IC_50_ > 100 µg/mL as inactive. Crude ethanolic extracts from Gaysorn-tang-ha, Trikatuk, and Triphala exhibited potent activity against *the P. falciparum* K1 strain with IC_50_ values of 2.81, 4.37, and 4.39 µg/mL, respectively. The ethanolic extract of Benjatian revealed moderate antiplasmodial activity (IC_50_ = 15.5 µg/mL), and all other extracts showed good activity with IC_50_ ranging from 6.8–8.5 µg/mL. On the other hand, most of the aqueous extracts showed no activity (IC_50_ > 5 µg/mL). Interestingly, aqueous extract from Jatu-phala-tiga had a potent activity with an IC_50_ of 5.0 µg/mL, and extracts from Triphala and Trisamo expressed good activity with IC_50_ of 5.7 and 6.1 µg/mL, respectively.

### 3.4. In Vitro Cytotoxicity on HepG2 and Vero Cells

The cytotoxic effects of the ten traditional recipes against HepG2 and Vero cells are shown in Table 4. A benchmark of toxicity levels based on the National Cancer Institute criteria was used. Recipes with CC_50_ values <30 µg/mL are considered cytotoxic after 48–72 h of exposure [27]. The results of PBS and 0.5% DMSO treated cells were considered 100% of living cells for aqueous and ethanolic extracts. The cytotoxic effects on HepG2 cells revealed that most of the extracts had CC_50_ values greater than the specified criteria. Only three extracts showed cytotoxicity with a CC_50_ of <30 μg/mL, indicating mild cytotoxicity [28]. The ethanolic extracts from Benjakul, Benjalotiga, and Trikatuk exhibited CC_50_ values of 10.9, 20.1, and 26.5 μg/mL, respectively. A comparison between the effects of aqueous and ethanolic extracts on HepG2 cells showed that the ethanolic extract had a greater toxic effect. The cytotoxic effects on Vero cells demonstrated that ethanolic extract from Chan-tang-ha is more toxic compared to that of the others, which produced CC_50_ value <12.5 μg/mL, and Benjalotiga had toxic effects with CC_50_ of 15.3 μg/mL for aqueous and 20.5 μg/mL for ethanolic extracts.

### 3.5. Interpretation of Selectivity Index (SI)

The SI was used to calculate risk-benefit assessment and identify progressible extracts that measure the ratio between 50% toxic concentration (CC_50_) and 50% antiplasmodial concentration (IC_50_) [29]. An extract with values <1 could be toxic and not applicable; however, a high value will give active extracts without undue risk. As shown in Table 4, both aqueous and ethanolic extracts of Triphala, Trisamo, and Jatu-phala-tiga showed high values of >27 in both HepG2 and Vero cells. The ethanolic extracts of Gaysorn-tang-ha exhibited high SI values above 20 in HepG2 cells, whereas the ethanolic extracts of Benjathian, Benjagot, and Chan-tang-ha showed high SI values in Vero cells. In addition, the ethanolic extract from other extracts exhibited values greater than that of the aqueous extract in both cell lines.

### 3.6. Hemolysis Results

**Figure 2:**
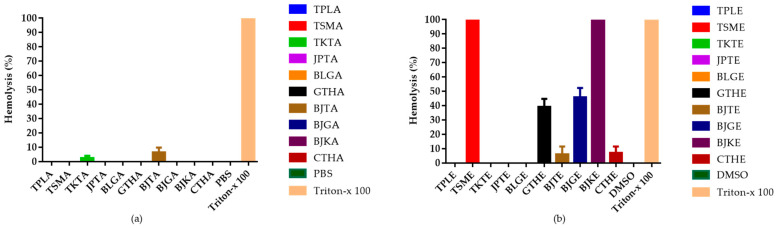
Percentage of hemolysis from in vitro hemolysis assay against human erythrocyte. (**a**) Hemolytic effects of aqueous extracts from ten recipes at 50 µg/mL; (**b**) Hemolytic effects of ethanolic extracts at 50 µg/mL concentration. TPLA, aqueous extracts from Triphala; TSMA, aqueous extracts from Trisamo; TKTA, aqueous extracts from Trikatuk; JPTA, aqueous extracts from Jatu-phala-tiga; BLGA, aqueous extracts from Benjalotiga; GTHA, aqueous extracts from Gaysorn-tang-ha; BJTA, aqueous extracts from Benjathian; BJGA, aqueous extracts from Benjagot; BJKA, aqueous extracts from Benjakul; CTHA, aqueous extracts from Chan-tang-ha; TPLE, ethanolic extracts from Triphala; TSME, ethanolic extracts from Trisamo; TKTE, ethanolic extracts from Trikatuk; JPTE, ethanolic extracts from Jatu-phala-tiga; BLGE, ethanolic extracts from Benjalotiga; GTHE, ethanolic extracts from Gaysorn-tang-ha; BJTE, ethanolic extracts from Benjathian; BJGE, ethanolic extracts from Benjagot; BJKE, ethanolic extracts from Benjakul; CTHE, ethanolic extracts from Chan-tang-ha; PBS, phosphate-buffered saline; and DMSO, dimethyl sulfoxide.

The hemolytic effects of the ten recipes were determined to indicate hematotoxicity in human erythrocytes, and the results are shown in Figure 2. Triton X-100 (0.5% *v*/*v*) was used for complete hemolysis of blood cells, which was then compared with the samples. Cell lysis was measured as the amount of free hemoglobin in the red cell suspension. Aqueous extracts at a fixed dose of 50 μg/mL did not show lytic effects on erythrocytes, except for Benjathian (6.53%) and Trikatuk (2.46%), whereas some ethanolic extracts showed hemolytic effects. The effect of Trisamo and Benjakul recipes reached 100% hemolysis after incubation for 72 h. Benjagot, Gaysorn-tang-ha, Chan-tang-ha, and Benjathian exhibited hemolysis percentages of 45.59, 39.18, 6.09, and 5.79, respectively.

### 3.7. Estimation of Intracellular Reactive Oxygen Species Production

**Figure 3:**
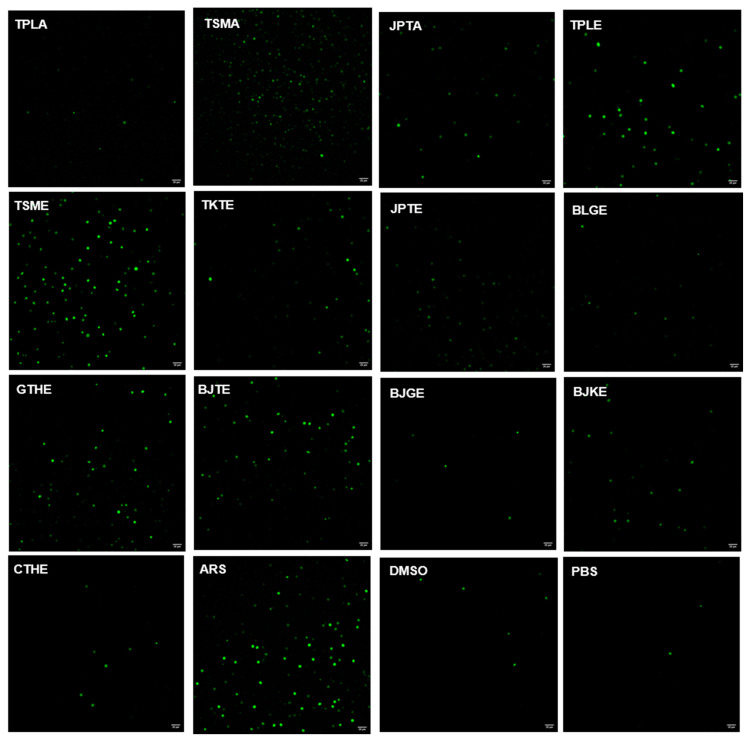
Evaluation of intracellular oxidant generation in *P. falciparum*-infected red blood cells using CM-H2DCFDA staining; Scale bar = 20 µm., TPLA, aqueous extracts from Triphala; TSMA, aqueous extracts from Trisamo; JPTA, aqueous extracts from Jatu-phala-tiga; TPLE, ethanolic extracts from Triphala; TSME, ethanolic ex-tracts from Trisamo; TKTE, ethanolic extracts from Trikatuk; JPTE, ethanolic extracts from Ja-tu-phala-tiga; BLGE, ethanolic extracts from Benjalotiga; GTHE, ethanolic extracts from Gaysorn-tang-ha; BJTE, ethanolic extracts from Benjathian; BJGE, ethanolic extracts from Benjagot; BJKE, ethanolic extracts from Benjakul; CTHE, ethanolic extracts from Chan-tang-ha; ARS, artesunate; PBS, phosphate-buffered saline; DMSO, dimethyl sulfoxide.

**Figure 4:**
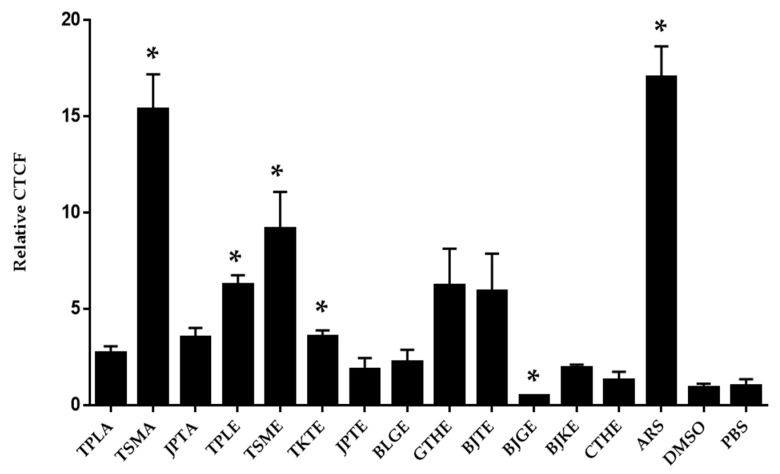
Detection of oxidant levels by confocal fluorescence microscopy. Data were analyzed by independent *t*-test. * *p* < 0.05 versus the negative control groups; TPLA, aqueous extracts from Triphala; TSMA, aqueous extracts from Trisamo; JPTA, aqueous extracts from Jatu-phala-tiga; TPLE, ethanolic extracts from Triphala; TSME, ethanolic extracts from Trisamo; TKTE, ethanolic extracts from Trikatuk; JPTE, ethanolic extracts from Jatu-phala-tiga; BLGE, ethanolic extracts from Benjalotiga; GTHE, ethanolic extracts from Gaysorn-tang-ha; BJTE, ethanolic extracts from Benjathian; BJGE, ethanolic extracts from Benjagot; BJKE, ethanolic extracts from Benjakul; CTHE, ethanolic extracts from Chan-tang-ha; ARS, artesunate; PBS, phosphate-buffered saline; DMSO, dimethyl sulfoxide.

CM-H2DCFDA was used to detect free radical production. The reaction of an oxidant and a CM-H2DCFDA non-fluorescent dye generates a DCF fluorescent component. Oxidant production is indicated by green fluorescence, as shown in Figure 3. Cells treated with DMSO or PBS were considered to have basal levels of oxidant generation. The positive control exhibited the highest oxidant production. The average CTCF values from positive control; aqueous extract of Trisamo; ethanolic extracts of Triphala, Trisamo, and Trikatuk recipes significantly increased ROS levels compared with the levels in negative groups at *p* < 0.05 by 17.06%, 15.36%, 6.26%, 9.20%, and 3.60%, respectively (Figure 4). Contrastingly, oxidant generation in the ethanolic extract group of the Benjagot recipe was significantly decreased by 0.50%.

### 3.8. Selection of Crude Extract as a Candidate in a Mouse Model

Table 4 shows the SI values of the 20 crude extracts on the HepG2 and Vero cell lines. Nine SI values that were not determined included the values of aqueous extracts of Gaysorn-tang-ha, Benjagot, and Benjakul on both HepG2 and Vero cells, and aqueous extracts of Trikatuk, Benjatian, and Chan-tang-ha on Vero cells, because SI values cannot be calculated. The aqueous extract of Triphala showed the highest values in HepG2 cells, whereas the ethanolic extract showed a high constant value of 176.13 in Vero cells. It is imperative to review the formula used to calculate SI indices. The values of IC_50_ produced the same antiplasmodial activity, which was 5.7 ± 0.2 for aqueous and 4.4 ± 1.3 μg/mL for ethanolic extracts, whereas the values of cytotoxicity aqueous extract were greater than ethanolic in both HepG2 and Vero cell lines. Therefore, an aqueous extract of Triphala was selected for testing in mouse models.

### 3.9. Chemical Profiling of Aqueous Extract of Triphala

**Figure 5:**
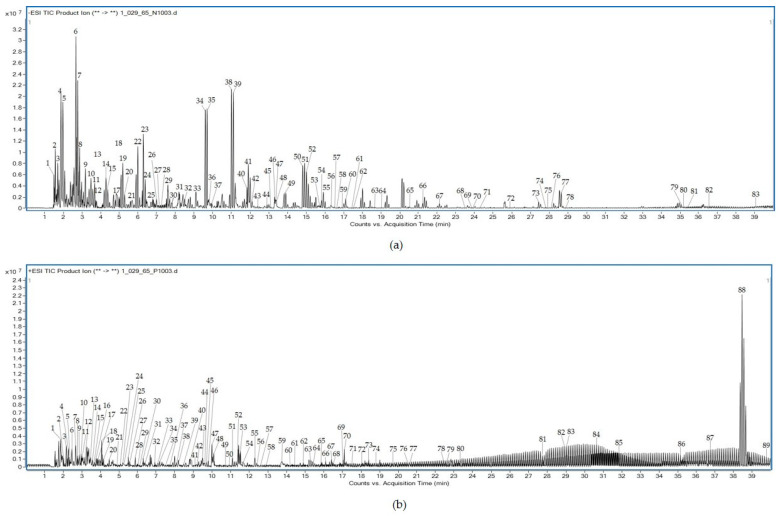
LC-QTOF-MS full-scan chromatogram of aqueous extract from Triphala in negative (**a**) and positive (**b**) modes.

**Figure 6:**
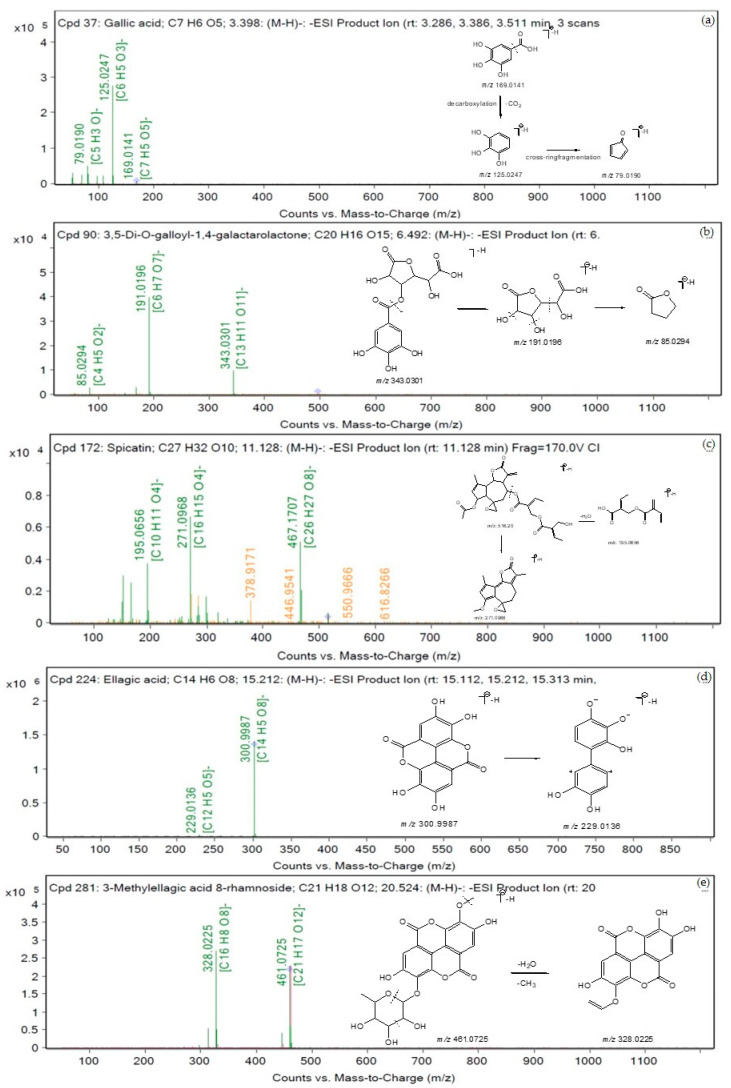
Proposed fragmentation patterns in negative mode (**a**) gallic acid, (**b**) 3,5-Di-O-galloyl-1,4-galactarolactone, (**c**) spicatin, (**d**) ellagic acid, and (**e**) 3-Methylellagic acid 8-rhamnoside.

**Figure 7:**
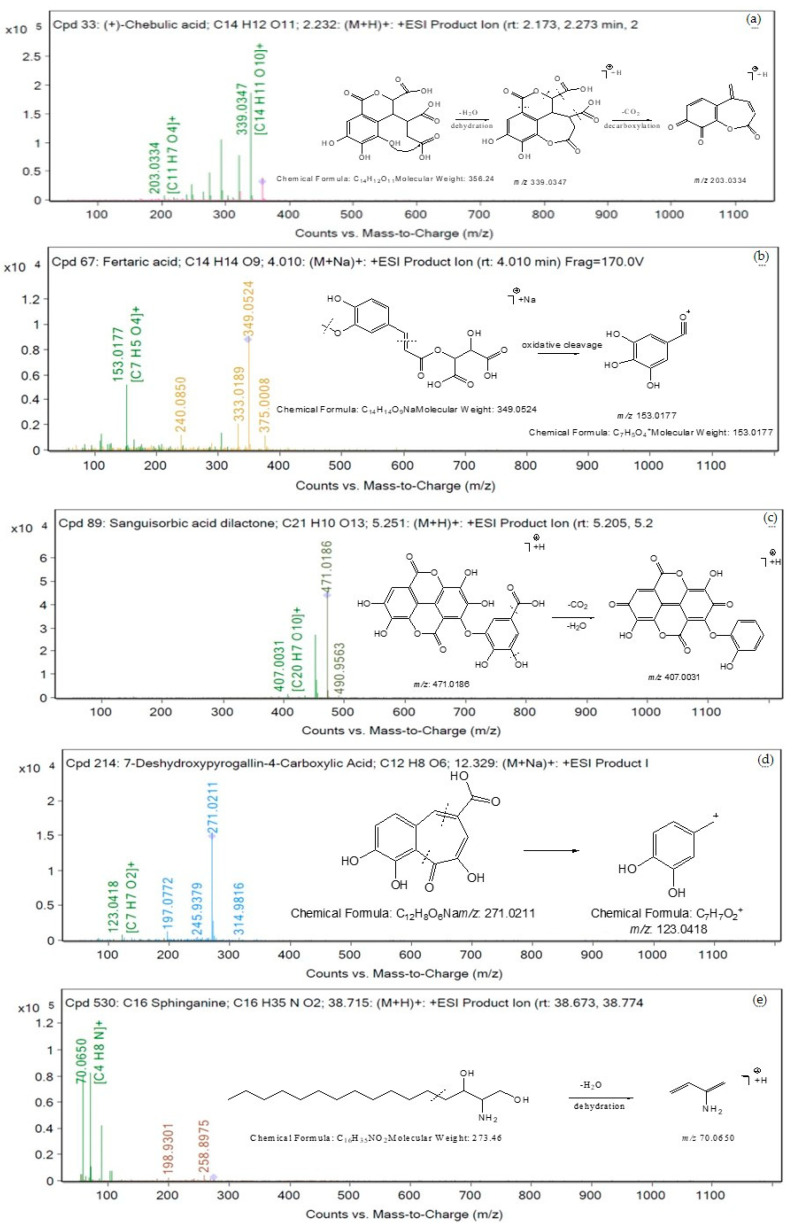
Proposed fragmentation patterns in positive mode (**a**) chebulic acid, (**b**) fertaric acid, (**c**) sanguisorbic acid dilactone, (**d**) 7-deshydroxypyrogallin-4-carboxylic acid, and (**e**) C16 Sphinganine.

A total of 83 metabolites in the negative ion mode and 89 metabolites in the positive ion mode were identified from the aqueous extract of Triphala using LC-QTOF-MS. Table 5 shows several compounds identified from the Mass Hunter METLIN database library by matching their accurate masses. The peak chromatograms are presented in Figure 5. Figure 6 and Figure 7 showed proposed fragmentation patterns in negative and positive modes, respectively.

### 3.10. Effects of Aqueous Extract of Triphala on 4-Day Suppressive Test

Table 6 shows the percentage of parasitemia and suppression in mice infected with *P. berghei*. Although the most effective suppression was exhibited in the chloroquine group, which produced 100% suppression, the administration of the extract at all doses revealed a significant (*p* < 0.05) increase in percent suppression when compared with that of the negative control. Different doses of the extract reduced the percentage of parasitemia in a dose-dependent manner. The extract at 600 mg/kg showed the best suppressive effect (75.47%), followed by 44.13% at the dose of 400 mg/kg and 42.72% at the dose of 200 mg/kg.

### 3.11. Acute Toxicity Test

The mice that received the aqueous extract of Triphala at 2 g/kg did not experience any death or abnormalities of the eyes, fur, or skin and did not show behavioral changes such as general activity, tremors, convulsions, ataxia, or diarrhea, indicating that the LD_50_ value of the extract was greater than 2 g/kg. Both groups of mice showed a percent increase in body weight, and no significant difference (*p* < 0.05) was observed between the two groups (Table 7). The relative organ weights of the liver and kidney in mice that received the extract showed no significant difference (*p* < 0.05) compared to the control group (Table 8). In addition, the extract did not induce significant (*p* < 0.05) changes in the liver and kidney enzymes, including BUN, creatinine, AST, ALT, and ALP (Table 9).

**Figure 8:**
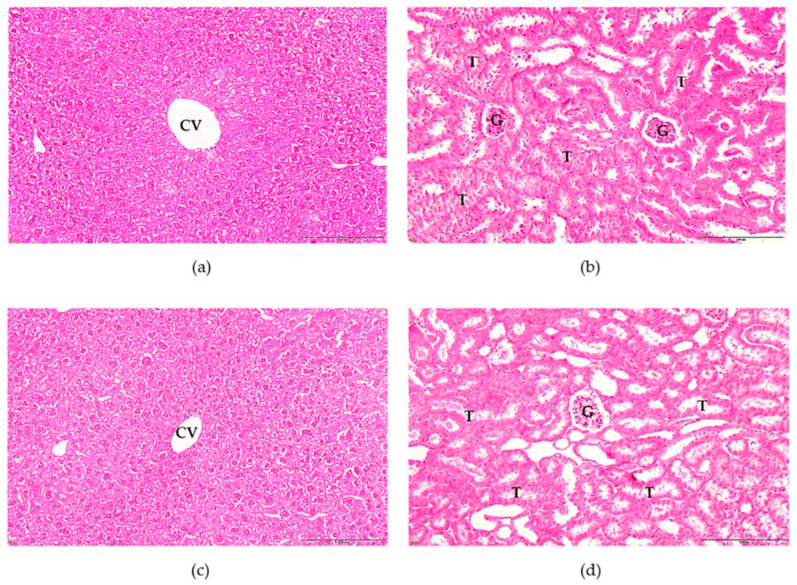
Histopathology of the liver and kidney from ICR mice that received aqueous extract from Triphala in acute toxicity test; (**a**) liver histology in control mice, (**b**) kidney histology in control mice, (**c**) liver histology in extract-treated mice and (**d**) kidney histology in extract-treated mice. All images were acquired at 20X magnification. Bar = 20 μm. CV, central vein; T, renal tubule; G, glomerulus.

Figure 8 shows the histological examination of the control and treatment groups, which showed normal structures of the liver and kidney in both groups. The liver sections did not show dilation of the central vein, cytoplasmic vacuolization, inflammatory cell infiltration, or congestion in the hepatic sinusoids. Kidney histological changes in extract-treated mice exhibited no obvious damage compared to the control group. Figure 8b,d show normal renal tubules and glomeruli in the glomerular basement membrane.

## 4. Discussion

Over the years, drug resistance has become one of the biggest problems in infectious diseases, including malaria. Partial resistance to artemisinin has emerged and spread, leading to delayed parasite clearance after treatment with ACT [30]. The development of antimalarial agents has been urgently needed; hence, phytomedicine became an interesting idea [31]. This study investigated the antiplasmodial activity of ten traditional recipes that provided scientific justification for malaria treatment. Solvent choices for extraction were selected according to the traditionally used phytomedicine in Thailand, which has otherwise been used as a polar and slightly nonpolar solvent for the study of biological activities. An aqueous solution is considered the best choice because of its low cost, nontoxicity, and health safety, and ethanol can be added to increase the solubilization of polar substances [32]. In addition, different solvent polarities affect the extract yields. The Benjalotiga and Chan-tang-ha recipes produced higher ethanolic yields than that of aqueous solutions. This result is in agreement with previous studies, where they found that the compounds present in trees can be extracted using organic solvents [33]. This finding suggests that alcohol can improve the solubilization of nonpolar molecules from wood [34].

Antiplasmodial properties of herbal recipes revealed that high activity against *P. falciparum* was mostly present in ethanolic extracts with an IC_50_ value ranging from 2.8–8.5 μg/mL. In comparison, the Gaysorn-tang-ha recipe showed the highest activity, followed by Trikatuk, Triphala, Benjalotiga, Benjagot, Chan-tang-ha, Jatu-phala-tiga, Benjakul, and Benjathian recipes. This suggests that the solubility of potentially active substances in semi-polar solutes may be greater than that in polar solvents, which is consistent with previous research. Potent antiplasmodial compounds are concentrated in the medium-polar solvent [35]. In addition, single ethanolic extracts from *Dracaena loureiri* Gagnep, *Myristica fragrans* Houtt, and *Piper chaba* Hunt were reported to possess antiplasmodial activity with IC_50_ of 10.5, 8.9, and 5.3 μg/mL, respectively [19,36]. In contrast, only three aqueous extracts from Triphala, Trisamo, and Jatu-phala-tiga exhibited good activity with an IC_50_ of 5.7 ± 0.2, 6.1 ± 0.7, and 5.0 ± 0.3 μg/mL, respectively, which is in agreement with previous evidence. The ingredients in water extracts of Triphala, Trisamo, and Jatu-phala-tiga recipes such as *Terminalia bellerica* (Gaertn) Roxb., *Phyllanthus emblica* L., and *Terminalia chebula* Retz were reported antiplasmodial activities with an IC_50_ of 14.3, 14.4, and 15.4 μg/mL, respectively [37]. Therefore, our findings imply that the antiplasmodial activity of these recipes is caused by the combined effect or synergism of the ingredients.

Historically, plants have been considered to have pharmacological properties that respond to the presence of phytocompounds [38]. This study investigated eight phytoconstituents based on the main components present in medicinal plants [39]. Flavonoids, a group of natural substances with aromatic organic structures, are potential sources of antimalarial compounds [40]. Its mechanism of action is believed to be its interference with functional biomolecules, such as protein, enzymes, DNA, etc., under cellular oxidative stress and inhibition of fatty acid biosynthesis during the intraerythrocytic cycle [40]. Terpenoids play a key role in the eradication of malarial parasites. Artemisinin, a sesquiterpene lactone compound, is currently the most effective antimalarial drug derived from the medicinal plant [41]. They create radical ions that can damage various proteins, including sarco-endoplasmic reticulum Ca^2+^-ATPase, and inhibit PfATP6, leading to the breakage of mitochondrial and parasitic membranes [42]. Alkaloids are a broad class of biological compounds with antiplasmodial properties. Quinoline alkaloids are well-known compounds in malaria research, such as quinine and quinidine. For quinine, the structure has been modified to improve efficacy and reduce toxicity. Its mechanism is related to hemoglobin breakdown pathways. [43,44]. Tannins have a positive effect on antiplasmodial prophylaxis [45,46]. Saponins and coumarins have been reported as attractive compounds against malaria [47,48]. Therefore, plant secondary metabolites are strongly correlated with antiplasmodial activities, and it is suggested that the good activity of the traditional recipes in this study may be owing to the action of one individual or synergistic effects of phytocompounds. Traditional herbal medicines not only provide benefits but also generate potentially harmful effects or side effects from the plants [49]. This encouraged us to investigate the toxic effects of these medicinal plants.

An in vitro cell-based approach was used as the model for toxicity screening. The liver is the primary site for drug-induced toxicity, whereas the kidney is the primary organ involved in drug clearance for oral drug delivery [50]. Consequently, we investigated the cytotoxic effects of ten traditional recipes on both Hep-G2 and Vero cells and found that all extracts showed CC_50_ against Hep-G2 at concentrations greater than 30 μg/mL, except for the ethanolic extracts from Benjakul, Benjalotiga, and Trikatuk, whereas Benjalotiga and the aqueous extract of Chan-tang-ha exhibited CC_50_ against Vero cells at concentrations below 30 μg/mL. These results suggest that the extracts with a CC_50_ below 30 μg/mL exhibited cytotoxicity. This finding is consistent with those of previous studies [51,52]. The ethanolic extract of Benjakul possesses anticarcinogenic activity, which is a response of at least three cytotoxic components of plumbagin, piperine, and 6-gingerol. Likewise, the toxic effects of Trikatuk may be caused by its components. For the Benjalotiga recipe, Santalol, the major constituent of *Santalum album* L., was reported to have antitumor properties on human hepatocellular carcinoma cell lines, and Silvestrol and episilvestrol were announced as potential anticancer agents against human oral epidermoid carcinoma [53,54]. Taccalonolides isolated from plants of the genus Tacca, such as *Tacca chantrieri*, are a new class of microtubule-stabilizing anticancer agents [55]. This finding suggests that the cytotoxicity of the recipes might be caused by toxic compounds that are deposited in plants. However, the toxic effects of these recipes on Vero cells exhibited CC_50_ greater than those on HepG2 cells, except for Benjalotiga. It was implied that ethanolic extracts from Trikatuk and Benjakul displayed selective toxicity towards cancer cell lines owing to the contribution of antitumor or anticancer compounds.

Furthermore, drug-induced hemolysis is a serious toxicity liability, particularly in malaria. The hemolytic toxicity of the recipes was quantified to forecast the direct harmful effects. Aqueous extracts except for Benjathian and Trikatuk at a fixed dose of 50 μg/mL did not promote the breakdown of red blood cells, and only three aqueous extracts from Triphala, Trisamo, and Jatu-phala-tiga exhibited high SI values. These results suggest that aqueous extracts from Triphala, Trisamo, and Jatu-phala-tiga are good candidates for further evaluation in animal models. In contrast, some ethanolic extracts exhibited hemolytic effects. Trisamo and Benjakul recipes produced 100% hemolysis after incubation for 72 h. Benjagot, Gaysorn-tang-ha, Chan-tang-ha, and Benjathian exhibited percent hemolysis at 45.59 ± 6.81, 39.18 ± 9.62, 6.09 ± 9.14, and 5.79 ± 9.66, respectively. The extracts may be attributed to the presence of toxic substances that affect hematopoietic cells. In addition, this finding may imply that the antiplasmodial activity of ethanolic extracts of Gaysorn-tang-ha, Benjagot, Chan-tang-ha, Trisamo, Benjakul and Benjatian with IC_50_ of 2.8, 6.8, 7.2, 7.7, 8.5 and 15.5 μg/mL, respectively could be due to hemolytic activity at 50 µg/mL.

Based on these findings, we propose that extracts not inducing hemolysis must be considered for use in medicine, whereas extracts that show hemolysis effects of more than 10% should be identified as toxic components or used with caution.

In previous findings, the antiparasitic effect of artesunate has been linked with DNA damage by increasing ROS production [56]. In our study, we observed oxidant generation of the extracts that had the ability to kill the parasite. As shown in Figure 3, our results illustrated that aqueous extracts of Trisamo and ethanolic extracts of Triphala, Trikatuk, and Trisamo induced significantly higher oxidant levels, suggesting that parasite death might be related to enhancing of oxidant levels and that increasing the oxidant can be targeted to oxidative damage to intracellular proteins, lipids and nucleic acids [57]. This finding is in accordance with previous research. Phenolic compounds in Terminalia species could potentially behave as either antioxidants or prooxidants, depending on their concentration, redox state, and the ratio between compounds [58]. The antioxidant effect is due to acting with a variety of free radicals, whereas prooxidant properties are related to the presence of transition metal ions such as copper or iron in the extract. The ability to reduce the metal ions may exert a redox cycling mechanism resulting in the formation of prooxidants [58].

In order to investigate the antimalarial activity and toxicity in an animal model, an aqueous extract of Triphala was selected for the in vivo evaluation. However, additional candidates such as Trisamo and Jatu-phala-tiga can be chosen as candidates in further studies because extracts with SI values of ≥10 can be assumed as potential samples for further investigation [59]. ICR mice were used because they are susceptible to infection by *P. berghei* ANKA [60]. The aqueous extract of Triphala reduced the parasite load up to 75.47% at a concentration of 600 mg/kg. Although the standard drug eliminated the infection, the extract at all doses significantly suppressed parasitemia (*p* < 0.05) compared to that of the infected control. This finding implies that the extract possesses good antimalarial activity against *P. berghei* ANKA. This activity may have been derived from the active compounds deposited in the extract. Gallic acid increases ROS production in macrophages, which may enhance phagocytic activity, and ellagic acid has antimalarial activity [61,62,63]. In addition, previous studies found gallic acid, ellagic acid, and chebulinic acid to be the major constituents of the Triphala recipe [11]. There was consistency with our results by LC-QTOF-MS analysis. Furthermore, Triphala has been used to maintain appropriate homeostasis in the body. Thus, the reduction in parasites might be owing to indirect effects. Triphala possesses antioxidant, free-radical scavenging, and immunomodulatory activities [63,64]. Therefore, the extract may activate the mechanism of cell-mediated immunity or humoral-mediated immunity because it is responsible for the stimulation of the immune system [63].

The harmful effects of the aqueous extract of Triphala showed that the extract did not cause acute toxicity in ICR mice. The indications for safety were normal behavior and absence of alteration in body weight, organ weight, liver-kidney function level, and histology compared with that of the control mice. Changes in body and organ weights are important parameters for assessing toxicity. Weight loss may indicate that a substance is causing damage to the body. Organ weight is a sensitive indicator of chemical or drug-induced organ damage [65]. Thus, the results of this study indicate that the extract is safe for the liver and kidneys. In addition, the results of liver-kidney functions and histological examination showed no significant changes compared to the control group. Therefore, we conclude that the aqueous extract of Triphala is safe.

## 5. Conclusions

This study confirmed that all ethanolic and aqueous extracts from Triphala, Trisamo, and Jatu-phala-tiga could kill *P. falciparum*. However, high SI values in both HepG2 and Vero cells were present in aqueous extracts from Triphala, Trisamo, and Jatu-phala-tiga recipes. The aqueous extract of Triphala exhibited good antimalarial activity in a mouse model, and a single oral dose of 2 g/kg was safe in acute toxicity tests.

## Figures and Tables

**Table 1:** List of traditional recipes.

Recipe	Plant Ingredients	Common Name	Plant Part	Family	Voucher Number
*Triphala*	*Terminalia bellirica* (Gaertn.) Roxb.	Beleric myrobalan	fruits	Combretaceae	SMD074002003
	*Terminalia chebula*	Chebulic myrobalan	fruits	Combretaceae	SMD070006007
	*Phyllanthus emblica*	Indian gooseberry	fruits	Phyllanthaceae	SMD209003007
Trikatuk	*Zingiber officinale*	Ginger	rhizome	Zingiberaceae	SMD288015005
	*Piper nigrum*	Black Pepper	fruits	Piperaceae	SMD209001014
	*Piper chaba Hunter*	Javanese long pepper	fruits	Piperaceae	SMD209002003
Trisamo	*Terminalia bellirica* (Gaertn.) Roxb.	Beleric myrobalan	fruits	Combretaceae	SMD074002003
	*Terminalia chebula*	Chebulic myrobalan	fruits	Combretaceae	SMD070006007
	*Terminalia arjuna* (Roxb. ex DC.)	Arjun	fruits	Combretaceae	SMD070006002
Jatu-phala-tiga	*Terminalia bellirica* (Gaertn.) Roxb.	Beleric myrobalan	fruits	Combretaceae	SMD074002003
	*Terminalia chebula*	Chebulic myrobalan	fruits	Combretaceae	SMD070006007
	*Phyllanthus emblica*	Indian gooseberry	fruits	Phyllanthaceae	SMD209003007
	*Terminalia arjuna* (Roxb. ex DC.)	Arjun	fruits	Combretaceae	SMD070006002
Benjalotiga	*Dracaena loureiroi* Gagnep.	Dragon blood	wood	Asparagaceae	SMD096001007
	*Santalum album* L.	Sandalwood	wood	Santalaceae	SMD210023002
	*Aglaia silvestris*	Kalament	wood	Meliaceae	SMD169052002
	*Tacca chantrieri*	Bat flower	wood	Dioscoreaceae	SMD095012002
	*Cyathea podophylla*	Tree fern	wood	Cyatheaceae	SMD116013001
Gaysorn-tang-ha	*Jasminum sambac* Ait	Arabian jasmine	flowers	Oleaceae	SMD187007002
	*Mimusops elengi* L.	Spanish cherry	flowers	Sapotaceae	SMD249006002
	*Mesua ferrea* L.	Ceylon ironwood	flowers	Calophyllaceae	SMD122007001
	*Nelumbo nucifera*	Sacred lotus	flowers	Nelumbonaceae	SMD181001001
	*Mammea siamensis*	Negkassar	flowers	Calophyllaceae	SMD122006002
Benjathian	*Nigella sativa* L.	Black cumin	seed	Ranunculaceae	SMD228005001
	*Lepidium sativum* L.	Garden cress	seed	Brassicaceae	SMD079003001
	*Cuminum cyminum* L.	Spice cumin	seed	Apiaceae	SMD017002001
	*Foeniculum vulgare* Miller subsp. var. vulgare	Common fennel	seed	Apiaceae	SMD017002002
	*Anethum graveolens* L.	Dill	seed	Apiaceae	SMD276001001
Benjagot	*Angelica dahurica* Hoffm. Benth. & Hook.f. ex Franch. & Sav	Dahurian angelica	rhizome	Apiaceae	SMD276002003
	*Atractylodes lancea* (Thunb.) DC.	Cang zhu	rhizome	Asteraceae	SMD072010001
	*Ligusticum sinense* Oliv.	Chuang xiong	rhizome	Apiaceae	SMD017003002
	*Angelica sinensis* (Oliv.) Diels	Dong quai	rhizome	Umbelliferae	SMD017003003
	*Artemisia vulgaris* L	Common mugwort	rhizome	Asteraceae	SMD029002004
Benjakul	*Piper chaba Hunter*	Javanese long pepper	fruits	Piperaceae	SMD209002003
	*Piper sarmentosum* Roxb.	Wildbetal leafbush	roots	Piperaceae	SMD213011002
	*Piper interruptum* Opiz.	Parsley panax	stem	Piperaceae	SMD213009001
	*Plumbago indica* L.	Indian leadwort	roots	Plumbaginaceae	SMD212004002
	*Zingiber officinale* Roscoe.	Ginger	rhizome	Zingiberaceae	SMD288015005
Chan-tang-ha	*Dracaena loureiroi* Gagnep.	Dragon blood	wood	Asparagaceae	SMD096001007
	*Tarenna hoaensis* Pitard	Kalamet	wood	Rubiaceae	SMD240002003
	*Santalum album* L.	Sandalwood	wood	Santalaceae	SMD210023002
	*Myristica fragrans* Houtt.	Nutmeg	wood	Myristicaceae	SMD177001003
	*Aglaia silvestris*	Kalament	wood	Meliaceae	SMD169052002

**Table 2:** Percentage extraction yields of aqueous and ethanolic extracts.

Recipe	Yield (%) (*w*/*w*)	
	Aqueous	Ethanolic
Triphala	38.58	20.12
Trikatuk	15.33	7.40
Trisamo	39.62	22.07
Jatu-phala-tiga	40.12	21.92
Benjalotiga	3.90	5.45
Gaysorn-tang-ha	19.22	6.15
Benjatian	16.50	2.07
Benjagot	37.47	6.45
Benjakul	7.33	4.35
Chan-tang-ha	3.97	7.52

**Table 3:** Phytochemical results of the ten traditional recipes (aqueous/ethanolic extracts).

Recipe	Secondary Metabolites (Aqueous/Ethanolic Extracts)							
	FL	TN	AL	TA	AN	CG	SA	CM
Triphala	+/−	+/+	+/+	+++/+++	−/−	−/−	+/−	−/−
Trikatuk	+/−	+/+	+/+	+++/−	−/−	−/−	+/−	−/+
Trisamo	+/−	+/+	−/−	+++/+++	−/−	−/−	++/+	−/−
Jatu-phala-tiga	+/−	+/+	+/+	++/++	−/−	−/−	−/−	−/−
Benjalotiga	+/+	+/+	+/+	+/−	−/−	−/−	+/−	+/+
Gaysorn-tang-ha	+/+	+/+	+/+	++/+	−/−	−/−	+/−	−/−
Benjatian	−/+	+/+	+/+	+/+	−/−	−/−	+/−	+/+
Benjagot	−/−	−/+	−/−	+/+	−/−	−/−	+/−	−/−
Benjakul	−/−	+/+	−/−	+/−	−/−	−/−	+/−	−/−
Chan-tang-ha	+/+	+/+	+/+	+/−	−/−	−/−	+/−	+/−

FL, flavonoids; TN, terpenoids; AL, alkaloids; TA, tannins; AN, anthraquinones; CG, cardiac glycosides; SA, saponins; CM, coumarins. +++, strong presence; ++, moderate presence; +, slight presence; −, absence.

**Table 4:** IC_50_ values for antiplasmodial activity, CC_50_ value against Hep G2 and Vero cell lines and selectivity index (SI) of ten traditional recipes.

Recipe Name	IC_50_ (µg/mL)		HepG2 CC_50_ (µg/mL)		Vero CC_50_ (µg/mL)	
	Aqueous	Ethanolic	Aqueous	Ethanolic	Aqueous	Ethanolic
Triphala	5.7 ± 0.2	4.4 ± 1.3	358.1 ± 9.7 ^64.5^	224.1 ± 5.5 ^51.1^	>800 ^>139.6^	773.2 ± 7.2 ^176.1^
Trikatuk	>100	4.4 ± 1.4	624.9 ± 1.9 ^<6.3^	26.5 ± 3.9 ^6.1^	>800 ^ND^	52.8 ± 3.1 ^12.1^
Trisamo	6.1 ± 0.7	7.7 ± 1.1	332.7 ± 12.2 ^54.5^	245.7 ± 12.0 ^31.9^	>800 ^>131.2^	>800 ^>103.8^
Jatu-phala-tiga	5.0 ± 0.3	7.4 ± 1.3	313.2 ± 1.5 ^63.3^	203.1 ± 6.4 ^27.5^	443.6 ± 11.1 ^89.6^	>800 ^>108.4^
Benjalotiga	>100	6.1 ± 1.6	231.1 ± 4.3 ^<3.2^	20.1 ± 3.0 ^3.3^	15.3 ± 2.5 ^<0.2^	20.5 ± 4.7 ^3.4^
Gaysorn-tang-ha	>100	2.8 ± 0.3	>800 ^ND^	60.3 ± 4.8 ^21.5^	>800 ^ND^	34.5 ± 4.7 ^12.3^
Benjatian	>100	15.5 ± 2.0	276.4 ± 9.0 ^<2.8^	71.1 ± 5.4 ^4.6^	>800 ^ND^	390.1 ± 7.2 ^25.1^
Benjagot	>100	6.8 ± 0.9	>800 ^ND^	30.0 ± 2.0 ^4.4^	>800 ^ND^	255.3 ± 8.9 ^37.5^
Benjakul	>100	8.5 ± 1.1	>800 ^ND^	10.9 ± 0.6 ^1.3^	>800 ^ND^	35.8 ± 2.6 ^4.2^
Chan-tang-ha	>100	7.2 ± 0.8	228.6 ± 4.8 ^<2.3^	65.5 ± 2.1 ^9.1^	<12.5 ^ND^	>800 ^>111.1^
Artesunate	3.9 ± 0.1 ng/mL		ND	ND	ND	ND
Doxorubicin	ND		0.9 ± 0.3		1.5 ± 0.0	

Data are presented as mean ± SEM; ND, not determined; Superscript values are selectivity index.

**Table 5:** Identification of the chemical constituents from aqueous extract from Triphala by LC-QTOF-MS.

No.	M/Z	RT (min)	Compounds	Formula	Molecular Weight
**Negative mode**					
1	283.2640	1.444	(+)-Isostearic acid	C_18_ H_36_ O_2_	284.2712
2	255.2327	1.532	Isopalmitic acid	C_16_ H_32_ O_2_	256.2400
3	181.0723	1.783	D-Sorbitol	C_6_ H_14_ O_6_	182.0795
4	209.0306	1.932	Galactaric acid	C_6_ H_10_ O_8_	210.0378
5	361.0415	2.045	2-O-Galloylgalactaric acid	C_13_ H_14_ O_12_	362.0488
6	331.0673	2.709	4-Glucogallic acid	C_13_ H_16_ O_10_	332.0746
7	355.0311	2.785	(+)-Chebulic acid	C_14_ H_12_ O_11_	356.0384
8	191.0202	2.935	Glucaric acid lactone	C_6_ H_8_ O_7_	192.0272
9	169.0146	3.398	Gallic acid	C_7_ H_6_ O_5_	170.0219
10	243.0513	3.536	1-O-Galloylglycerol	C_10_ H_12_ O_7_	244.0586
11	343.0308	3.749	5-O-Galloyl-1,4-galactarolactone	C_13_ H_12_ O_11_	344.0380
12	325.0565	3.887	Fertaric acid	C_14_ H_14_ O_9_	326.0638
13	191.0349	4.288	5,7-Dihydroxy-4-Methylcoumarin	C_10_ H_8_ O_4_	192.0422
14	265.0353	4.438	2-O-p-Coumaroyltartronic acid	C_12_ H_10_ O_7_	266.0427
15	133.0145	4.451	Malic acid	C_4_ H_6_ O_5_	134.0218
16	669.0942	4.551	Myricetin 3,7-diglucuronide	C_27_ H_26_ O_20_	670.1013
17	299.0409	4.764	Mumefural	C_12_ H_12_ O_9_	300.0482
18	213.0401	5.014	2-(1h-1,2,4-triazol-5-yl)-1h-isoindole-1,3(2h)-dione	C_10_ H_6_ N_4_ O_2_	214.0474
19	469.0046	5.165	Sanguisorbic acid dilactone	C_21_ H_10_ O_13_	470.0119
20	181.0145	5.340	2-Hydroxyisophthalic acid	C_8_ H_6_ O_5_	182.0217
21	313.0564	5.641	Salicyl phenolic glucuronide	C_13_ H_14_ O_9_	314.0637
22	317.0663	6.242	Dihydroisorhamnetin	C_16_ H_14_ O_7_	318.0736
23	495.0412	6.492	3,5-Di-O-galloyl-1,4-galactarolactone	C_20_ H_16_ O_15_	496.0484
24	359.0983	6.493	6′-Methoxypolygoacetophenoside	C_15_ H_2_0 O_10_	360.1055
25	541.0259	6.994	Punicacortein D	C_48_ H_28_ O_30_	1084.066
26	1083.0578	7.019	Punicalagin	C_48_ H_28_ O_30_	1084.065
27	347.0773	7.244	alpha-(1,2-Dihydroxyethyl)-1,2,3,4-tetrahydro-7-hydroxy-9-methoxy-3,4-dioxocyclopenta[c] [1]benzopyran-6-acetaldehyde	C_17_ H_16_ O_8_	348.0845
28	483.0782	7.370	1,2′-Di-O-galloylhamamelofuranose	C_20_ H_2_0 O_14_	484.0853
29	220.0615	7.845	Methyl dioxindole-3-acetate	C_11_ H_11_ N O_4_	221.0688
30	321.0251	7.996	Digallate	C_14_ H_10_ O_9_	322.0324
31	1083.1160	8.147	Putranjivain A	C_46_ H_36_ O_31_	1084.1230
32	467.1190	8.472	Leucodelphinidin 3-O-alpha-L-rhamnopyranoside	C_21_ H_24_ O_12_	468.1262
33	635.0888	9.099	3-O-Galloylhamamelitannin	C_27_ H_24_ O_18_	636.0959
34	651.0835	9.449	Amlaic acid	C_27_ H_24_ O_19_	652.0908
35	477.0671	9.474	Quercetin 3′-O-glucuronide	C_21_ H_18_ O_13_	478.0744
36	461.1660	9.825	Verbasoside	C_20_ H_30_ O_12_	462.1732
37	633.0744	10.001	Pterocaryanin B	C_27_ H_22_ O_18_	634.0813
38	785.0833	11.078	Sanguiin H1	C_34_ H_26_ O_22_	786.0905
39	515.1916	11.128	Spicatin	C_27_ H_32_ O_10_	516.1988
40	935.0783	11.729	1-O-Galloylpedunculagin	C_41_ H_28_ O_26_	936.0853
41	371.0979	11.854	Dihydroferulic acid 4-O-glucuronide	C_16_ H_2_0 O_10_	372.1052
42	247.0250	12.218	7-Deshydroxypyrogallin-4-Carboxylic Acid	C_12_ H_8_ O_6_	248.0323
43	447.093	12.431	1,2,6,8-Tetrahydroxy-3-methylanthraquinone 2-O-b-D-glucoside	C_21_ H_2_0 O_11_	448.1002
44	600.9889	12.807	Diellagilactone	C_28_ H_10_ O_16_	601.9960
45	465.1034	13.257	(-)-Epicatechin 7-O-glucuronide	C_21_ H_22_ O_12_	466.1105
46	197.0457	13.320	3,4-O-Dimethylgallic acid	C_9_ H_10_ O_5_	198.0530
47	473.0355	13.371	m-Trigallic acid	C_21_ H_14_ O_13_	474.0428
48	119.0501	13.558	Lentialexin	C_8_ H_8_ O	120.0575
49	953.0896	13.709	Isoterchebin	C_41_ H_30_ O_27_	954.0966
50	787.0995	14.711	1,2′,3,5-Tetra-O-galloylhamamelofuranose	C_34_ H_28_ O_22_	788.1065
51	431.0979	14.936	Isovitexin	C_21_ H_2_0 O_10_	432.1051
52	300.9993	15.212	Ellagic acid	C_14_ H_6_ O_8_	302.0065
53	421.0776	15.826	Isomangiferin	C_19_ H_18_ O_11_	422.0847
54	463.0878	15.939	Quercetin 3-galactoside	C_21_ H_2_0 O_12_	464.0951
55	491.0826	15.989	Isorhamnetin 4′-O-glucuronide	C_22_ H_2_0 O_13_	492.0898
56	357.1186	16.465	Phlorisobutyrophenone 2-glucoside	C_16_ H_22_ O_9_	358.1259
57	303.0509	16.515	(±)-Taxifolin	C_15_ H_12_ O_7_	304.0582
58	955.1046	16.666	Chebulinic acid	C_41_ H_32_ O_27_	956.1117
59	331.0819	16.866	2′,3,5-Trihydroxy-5′,7-dimethoxyflavanone	C_17_ H_16_ O_7_	332.0891
60	355.1027	17.116	1-O-2′-Hydroxy-4′-methoxycinnamoyl-b-D-glucose	C_16_ H_2_0 O_9_	356.1101
61	261.0406	17.417	2-Acetyl-5,8-dihydroxy-3-methoxy-1,4-naphthoquinone	C_13_ H_10_ O_6_	262.0478
62	435.0931	17.492	Taxifolin 3-arabinoside	C_20_ H_2_0 O_11_	436.1003
63	207.0661	18.682	Sinapyl aldehyde	C_11_ H_12_ O_4_	208.0734
64	259.0246	19.008	Urolithin D	C_13_ H_8_ O_6_	260.0320
65	461.0723	20.524	3-Methylellagic acid 8-rhamnoside	C_21_ H_18_ O_12_	462.0796
66	217.0503	21.301	Piperic acid	C_12_ H_10_ O_4_	218.0576
67	431.0975	22.178	Kaempferol 4′-rhamnoside	C_21_ H_2_0 O_10_	432.1048
68	571.1813	23.531	Amorphigenin O-glucoside	C_29_ H_32_ O_12_	572.1886
69	573.0874	23.881	Mangiferin 6′-gallate	C_26_ H_22_ O_15_	574.0948
70	303.0508	23.982	Pratenol B	C_15_ H_12_ O_7_	304.0580
71	673.2130	24.333	Premithramycin A2′	C_33_ H_38_ O_15_	674.2201
72	461.1088	25.961	Rhamnetin 3-rhamnoside	C_22_ H_22_ O_11_	462.1160
73	285.0404	27.490	Luteolin	C_15_ H_10_ O_6_	286.0478
74	301.0356	27.841	Hieracin	C_15_ H_10_ O_7_	302.0428
75	723.1920	27.916	Kaempferol 3-(3′’-p-coumaroylrhamnoside)-7-rhamnoside	C_36_ H_36_ O_16_	724.1991
76	367.1181	28.166	Glicoricone	C_21_ H_2_0 O_6_	368.1254
77	567.1135	28.767	Chrysophanol 8-(6-galloylglucoside)	C_28_ H_24_ O_13_	568.1207
78	329.0301	28.943	2,8-Di-O-methylellagic acid	C_16_ H_10_ O_8_	330.0374
79	287.2225	35.182	9,10-dihydroxy-hexadecanoic acid	C_16_ H_32_ O_4_	288.2297
80	503.3374	35.257	(3beta,19alpha)-3,19,23,24-Tetrahydroxy-12-oleanen-28-oic acid	C_30_ H_48_ O_6_	504.3446
81	273.0403	35.445	1,3,6-Trihydroxy-5-methoxyxanthone	C_14_ H_10_ O_6_	274.0475
82	343.0459	36.585	Aflatoxin GM1	C_17_ H_12_ O_8_	344.0531
83	401.1601	39.115	6-Hydroxy-9,9-dimethyl-5-(3-methyl-1-oxobutyl)-1-propyl-3H,9H-[1,2]-dioxolo [3′,4′:4,5]furo [2,3-f][1]benzopyran-3-one	C_22_ H_26_ O_7_	402.1674
**Positive mode**					
1	260.113	1.930	Osmaronin	C_11_ H_17_ N O_6_	259.1059
2	401.012	2.044	2-O-Galloylgalactaric acid	C_13_ H_14_ O_12_	362.0488
3	357.0454	2.232	(+)-Chebulic acid	C_14_ H_12_ O_11_	356.0381
4	182.0813	2.382	L-Tyrosine	C_9_ H_11_ N O_3_	181.0739
5	136.0757	2.407	2-Phenylacetamide	C_8_ H_9_ N O	135.0685
6	355.0639	2.607	4-Glucogallic acid	C_13_ H_16_ O_10_	332.0749
7	180.1022	2.745	Phenacetine	C_10_ H_13_ N O_2_	179.0949
8	298.0922	2.883	Hexahydro-6,7-dihydroxy-5-(hydroxymethyl)-3-(2-hydroxyphenyl)-2H-pyrano [2,3-d]oxazol-2-one	C_13_ H_15_ N O_7_	297.085
9	367.0275	2.908	5-O-Galloyl-1,4-galactarolactone	C_13_ H_12_ O_11_	344.0381
10	339.1055	2.983	Hydroxytyrosol 1-O-glucoside	C_14_ H_20_ O_8_	316.1164
11	328.1392	3.083	N-(1-Deoxy-1-fructosyl)phenylalanine	C_15_ H_21_ N O_7_	327.1319
12	171.029	3.158	Gallic acid	C_7_ H_6_ O_5_	170.0217
13	260.0918	3.534	Skimmianine	C_14_ H_13_ N O_4_	259.0846
14	267.0478	3.610	1-O-Galloylglycerol	C_10_ H_12_ O_7_	244.0586
15	315.1053	3.710	Pantoyllactone glucoside	C_12_ H_20_ O_8_	292.116
16	335.0373	3.735	Cis-Caffeoyl tartaric acid	C_13_ H_12_ O_9_	312.0484
17	381.1175	3.885	202-791	C_17_ H_18_ N_4_ O_5_	358.1282
18	349.0532	4.010	Fertaric acid	C_14_ H_14_ O_9_	326.0638
19	327.0709	4.035	Sinapoyltartronate	C_14_ H_14_ O_9_	326.0637
20	433.1467	4.411	Butyl 3-O-caffeoylquinate	C_20_ H_26_ O_9_	410.1575
21	267.0839	4.887	threo-Syringoylglycerol	C_11_ H_16_ O_6_	244.0947
22	383.1311	5.138	2′-Methoxy-3-(2,4-dihydroxyphenyl)-1,2-propanediol 4′-glucoside	C_16_ H_24_ O_9_	360.142
23	471.0193	5.251	Sanguisorbic acid dilactone	C_21_ H_10_ O_13_	470.0119
24	235.0579	5.338	3-Hydroxy-4-methoxyphenyllactic acid	C_10_ H_12_ O_5_	212.0686
25	533.1263	5.539	Coenzyme F420-0	C_19_ H_22_ N_3_ O_12_ P	515.0924
26	507.0747	5.614	1,2′-Di-O-galloylhamamelofuranose	C_20_ H_20_ O_14_	484.0853
27	307.152	5.940	Dihydroartemisinin	C_15_ H_24_ O_5_	284.1627
28	427.121	6.216	Oleoside 11-methyl ester	C_17_ H_24_ O_11_	404.1318
29	294.0949	6.341	Deidaclin	C_12_ H_17_ N O_6_	271.1055
30	383.0944	6.541	6′-Methoxypolygoacetophenoside	C_15_ H_20_ O_10_	360.1051
31	367.1499	6.616	N-(1-Deoxy-1-fructosyl)tryptophan	C_17_ H_22_ N_2_ O_7_	366.1426
32	210.1128	6.842	Propoxur	C_11_ H_15_ N O_3_	209.1055
33	371.0735	7.218	alpha-(1,2-Dihydroxyethyl)-1,2,3,4-tetrahydro-7-hydroxy-9-methoxy-3,4-dioxocyclopenta[c] [1]benzopyran-6-acetaldehyde	C_17_ H_16_ O_8_	348.0843
34	223.1805	7.356	Noruron	C_13_ H_22_ N_2_ O	222.1732
35	323.1602	7.543	Zanthodioline	C_16_ H_19_ N O_5_	305.1264
36	369.1158	7.719	Aucubin	C_15_ H_22_ O_9_	346.1266
37	403.1365	8.120	Methyl helianthenoate A glucoside	C_19_ H_24_ O_8_	380.1472
38	373.1258	8.296	1,5-Dibutyl methyl hydroxycitrate	C_15_ H_26_ O_8_	334.1627
39	293.0995	8.496	Idebenone Metabolite (Benzenebutanoic acid, 2,5-dihydroxy-3,4-dimethoxy-6-methyl-)	C_13_ H_18_ O_6_	270.1105
40	217.0974	8.746	L-1,2,3,4-Tetrahydro-beta-carboline-3-carboxylic acid	C_12_ H_12_ N_2_ O_2_	216.09
41	417.1521	9.298	Gibberellin A43	C_20_ H_26_ O_8_	394.1627
42	216.0636	9.398	4-Hydroxy-5-phenyltetrahydro-1,3-oxazin-2-one	C_10_ H_11_ N O_3_	193.0744
43	231.1129	9.473	4-(N-Maleimido)phenyltrimethylammonium	C_13_ H_15_ N_2_ O_2_	231.1135
44	244.097	9.699	N-Desmethyltolmetin	C_14_ H_13_ N O_3_	243.0897
45	279.1202	9.849	2-[4-(3-Hydroxypropyl)-2-methoxyphenoxy]-1,3-propanediol	C_13_ H_20_ O_5_	256.1312
46	439.1578	9.849	Phenylethyl primeveroside	C_19_ H_28_ O_10_	416.1684
47	657.07	10.112	Pterocaryanin B	C_27_ H_22_ O_18_	634.0807
48	469.1324	10.124	Lucuminic acid	C_19_ H_26_ O_12_	446.143
49	373.1258	10.626	Fluprostenol Lactone Diol	C_18_ H_19_ F_3_ O_5_	372.1184
50	415.1361	10.826	Vermiculine	C_20_ H_24_ O_8_	392.1469
51	539.1891	11.152	Spicatin	C_27_ H_32_ O_10_	516.1997
52	337.0897	11.302	2-O-Acetylarbutin	C_14_ H_18_ O_8_	314.1004
53	659.0858	11.515	3-O-Galloylhamamelitannin	C_27_ H_24_ O_18_	636.0965
54	185.1074	11.753	Harmalan	C_12_ H_12_ N_2_	184.1002
55	271.0216	12.329	7-Deshydroxypyrogallin-4-Carboxylic Acid	C_12_ H_8_ O_6_	248.0323
56	277.0345	12.355	GW 9662	C_13_H_9_ClN_2_O_3_	276.0273
57	449.1074	12.455	Aureusidin 6-O-glucoside	C_21_ H_20_ O_11_	448.1002
58	153.0547	12.931	1-(2-Furanyl)-1,3-butanedione	C_8_ H_8_ O_3_	152.0475
59	785.0829	13.758	Granatin A	C_34_ H_24_ O_22_	784.0755
60	371.1104	14.309	Machaerol C	C_18_ H_20_ O_7_	348.1211
61	465.1364	14.559	1-O-E-Cinnamoyl-(6-arabinosylglucose)	C_20_ H_26_ O_11_	442.1472
62	443.1679	14.911	Citreoviridinol A1	C_22_ H_28_ O_8_	420.1786
63	303.0139	15.311	Ellagic acid	C_14_ H_6_ O_8_	302.0065
64	401.1571	15.336	Gibberellin A102	C_20_ H_26_ O_7_	378.1678
65	291.1567	15.888	Bisacurone epoxide	C_15_ H_24_ O_4_	268.1677
66	537.1729	16.063	Icariside II	C_27_ H_30_ O_10_	514.1837
67	355.173	16.414	(2E,4E,7R)-2,7-Dimethyl-2,4-octadiene-1,8-diol 8-O-b-D-glucopyranoside	C_16_ H_28_ O_7_	332.1836
68	335.1101	16.501	3-Hydroxychavicol 1-glucoside	C_15_ H_20_ O_7_	312.1208
69	355.1728	16.915	(4R,6S)-p-Menth-1-ene-4,6-diol 4-glucoside	C_16_ H_28_ O_7_	332.1834
70	333.0947	17.128	1-Pentadecanecarboxylic acid	C_15_ H_18_ O_7_	310.1054
71	545.1988	17.466	Isolariciresinol 4′-O-beta-D-glucoside	C_26_ H_34_ O_11_	522.2096
72	445.1831	18.105	Valtratum	C_22_ H_30_ O_8_	422.1939
73	397.1262	18.481	Rubone	C_20_ H_22_ O_7_	374.1368
74	401.1207	18.844	Hydroxyvernolide	C_19_ H_22_ O_8_	378.1315
75	395.1464	19.771	Hydroxymyricanone	C_21_ H_24_ O_6_	372.1569
76	463.0868	20.573	5,7,8,2′-Tetrahydroxyflavone 7-glucuronide	C_21_ H_18_ O_12_	462.0795
77	487.1939	20.648	Estriol-17-glucuronide	C_24_ H_32_ O_9_	464.2046
78	413.157	22.603	Rosmic acid	C_21_ H_26_ O_7_	390.1678
79	689.3873	22.891	Cyclopassifloside VI	C_36_ H_58_ O_11_	666.3978
80	383.1466	23.317	Lariciresinol	C_20_ H_24_ O_6_	360.1573
81	395.1465	27.877	Deguelin(-)	C_23_ H_22_ O_6_	394.1392
82	425.1936	28.943	Virolongin B	C_23_ H_30_ O_6_	402.2044
83	331.0445	29.093	2,8-Di-O-methylellagic acid	C_16_ H_10_ O_8_	330.0372
84	395.1821	30.797	16-phenyl-tetranor-PGE2	C_22_ H_28_ O_5_	372.1928
85	425.1938	31.950	Cortisone acetate	C_23_ H_30_ O_6_	402.2045
86	527.3348	35.282	Myrianthic acid	C_30_ H_48_ O_6_	504.3454
87	312.1595	36.810	1-Methoxy-4-[5-(4-methoxyphenoxy)-3-penten-1-ynyl]benzene	C_19_ H_18_ O_3_	294.1256
88	274.2746	38.715	C16 Sphinganine	C_16_ H_35_ N O_2_	273.2673
89	230.2482	38.941	Xestoaminol C	C_14_ H_31_ N O	229.2409

**Table 6:** In vivo antimalarial activity of aqueous extract from Triphala.

Group	Dose (mg/kg)	% Parasitemia	% Suppression
PBS	-	18.93 ± 0.81	-
Chloroquine	25	0	100 ^a^
Triphala (TPLA)	200	10.84 ± 0.30	42.72 ± 1.60 ^a, b, e^
	400	10.58 ± 0.20	44.13 ± 1.07 ^a, b, e^
	600	4.64 ± 0.95	75.47 ± 5.00 ^a, b, c, d^

Data are presented as mean ± SEM (n = 5 per group). Differences were considered statistically significant at *p* < 0.05. ^a^ Compared with the negative control group receiving PBS, ^b^ Compared with the positive control group receiving chloroquine, ^c^ Compared with TPLA 200, ^d^ Compared with TPLA 400, ^e^ Compared with TPLA 600.

**Table 7:** Effect of aqueous extract from Triphala on body weight changes in acute toxicity test.

Group	Body Weight (g)		% Increase in Body Weight
	Day 0	Day 14	
PBS	37.71 ± 0.56	41.68 ± 0.87	10.54 ± 1.51
Triphala	37.15 ± 0.72	41.04 ± 0.97	10.48 ± 1.44

All values are expressed as the mean ± SEM. There were no statistically significant differences at *p* < 0.05.

**Table 8:** Relative weight of liver and kidney in acute toxicity test of aqueous extract from Triphala.

Group	Relative Organ Weight	
	Liver	Kidney
PBS	6.96 ± 0.73	1.89 ± 0.12
Triphala	5.84 ± 0.15	1.86 ± 0.12

All values are expressed as the mean ± SEM. There were no statistically significant differences at *p* < 0.05.

**Table 9:** Effect of aqueous extract from Triphala on liver and kidney functions.

Group	BUN (mg/dL)	CREA (mg/dL)	AST (U/L)	ALT (U/L)	ALP (U/L)
PBS	24.60 ± 0.40	0.16 ± 0.00	119.80 ± 11.37	32.00 ± 1.97	124.40 ± 15.79
Triphala	23.20 ± 0.49	0.17 ± 0.01	97.00 ± 20.52	33.60 ± 1.47	101.00 ± 6.23

All values are expressed as the mean ± SEM. There were no statistically significant differences between groups at *p* < 0.05. BUN, blood urea nitrogen; CREA, creatinine enzyme; AST, aspartate aminotransferase; ALT, alanine transaminase; ALP, alkaline phosphatase.

## Data Availability

The data associated with this study are included in this published article. Additional files are available from the corresponding authors upon request.

## References

[1] World Malaria Report. https://www.who.int/teams/global-malaria-programme/reports/world-malaria-report-2021.

[2] Weatherall D.J., Miller L.H., Baruch D.I., Marsh K., Doumbo O.K., Casals-Pascual C., Roberts D.J. (2002). Malaria and the red cell. Hematology.

[3] Lee W.-C., Russell B., Rénia L. (2019). Sticking for a cause: The falciparum malaria parasites cytoadherence paradigm. Front. Immunol..

[4] WHO (2020). Report on Antimalarial Drug Efficacy, Resistance and Response: 10 Years of Surveillance.

[5] Thriemer K., Hong N.V., Rosanas-Urgell A., Phuc B.Q., Ha D.M., Pockele E., Guetens P., Van N.V., Duong T.T., Amambua-Ngwa A. (2014). Delayed parasite clearance after treatment with dihydroartemisinin-piperaquine in *Plasmodium falciparum* malaria patients in central Vietnam. Antimicrob Agents Chemother.

[6] Junsongduang A., Kasemwan W., Lumjoomjung S., Sabprachai W., Tanming W., Balslev H. (2020). Ethnomedicinal knowledge of traditional healers in Roi Et, Thailand. Plants.

[7] Noronha M., Pawar V., Prajapati A., Subramanian R.B. (2020). A literature review on traditional herbal medicines for malaria. S. Afr. J. Bot..

[8] Peltzer K., Pengpid S. (2019). The use of herbal medicines among chronic disease patients in Thailand: A cross-sectional survey. J. Multidiscip. Healthc..

[9] Parasuraman S., Thing G.S., Dhanaraj S.A. (2014). Polyherbal formulation: Concept of ayurveda. Pharmacogn. Rev..

[10] Rudrapal M., Celik I., Khan J., Ansari M.A., Alomary M.N., Alatawi F.A., Yadav R., Sharma T., Tallei T.E., Pasala P.K. (2022). Identification of bioactive molecules from Triphala (Ayurvedic herbal formulation) as potential inhibitors of SARS-CoV-2 main protease (Mpro) through computational investigations. J. King Saud Univ. Sci..

[11] Peterson C.T., Denniston K., Chopra D. (2017). Therapeutic uses of Triphala in Ayurvedic medicine. J. Altern. Complement. Med..

[12] Tappayuthpijarn P., Sattaponpan C., Sakpakdeecharoen I., Ittharat A. (2012). Cholinesterase inhibitory and antioxidant activities of Thai traditional remedies potentially used for Alzheimer’s disease. Eur. J. East Asian Stud..

[13] Pattanacharoenchai N., Itharat A. (2017). Anti-allergic activity of Trikatuk Tripha and Trisarn remedies. TMJ.

[14] Suksaeree J., Monton C. (2021). Evaluation of the interaction of phenolic compounds contained in the Trisamo recipe using simplex lattice design. J. Sci. Technol..

[15] Wetchakul P., Goon J.A., Adekoya A.E., Olatunji O.J., Ruangchuay S., Jaisamut P., Issuriya A., Kunworarath N., Limsuwan S., Chusri S. (2019). Traditional tonifying polyherbal infusion, Jatu-Phala-Tiga, exerts antioxidant activities and extends lifespan of *Caenorhabditis elegans*. BMC Complement. Altern. Med..

[16] Makchuchit S., Rattarom R., Itharat A. (2017). The anti-allergic and anti-inflammatory effects of Benjakul extract (a Thai traditional medicine), its constituent plants and its some pure constituents using in vitro experiments. Biomed. Pharmacother..

[17] Loetthammasak P. (2014). Formulas of Thai Traditional Medicine.

[18] Sinsupan N. (2002). Thai traditional medicine theory Part 3 Thai Pharmacy. J. Acad. Serv. Cent..

[19] Chaniad P., Phuwajaroanpong A., Techarang T., Horata N., Chukaew A., Punsawad C. (2022). Evaluation of the antimalarial activity and toxicity of Mahanil-Tang-Thong formulation and its plant ingredients. BMC Complement. Med. Ther..

[20] Ngbolua K.-T.-N. (2014). Phytochemical screening of some medicinal plants traditionally used by African women in Kinshasa city (DR Congo) for their intimate hygiene and evaluation of the pH of derived recipes. J. Mod. Drug Discov. Drug Deliv. Res..

[21] Malar G., Chinnachamy C. (2017). Phytochemical screening, total flavonoid, total terpenoid and anti-inflammatory activity of aqueous stem extract of *Salacia oblonga*. J. Chem. Pharm..

[22] Trager W., Jensen J.B. (2005). Human malaria parasites in continuous culture. 1976. J. Parasitol..

[23] Makler M.T., Hinrichs D.J. (1993). Measurement of the lactate dehydrogenase activity of *Plasmodium falciparum* as an assessment of parasitemia. Am. J. Trop. Med. Hyg..

[24] Peters W. (1975). The chemotherapy of rodent malaria, XXII. The value of drug-resistant strains of *P. berghei* in screening for blood schizontocidal activity. Ann. Trop. Med. Parasitol..

[25] Phuwajaroanpong A., Chaniad P., Horata N., Muangchanburee S., Kaewdana K., Punsawad C. (2020). In vitro and in vivo antimalarial activities and toxicological assessment of *Pogostemon Cablin* (Blanco) Benth. J. Evid.-Based Integr. Med..

[26] Lusakibanza M., Mesia G., Tona G., Karemere S., Lukuka A., Tits M., Angenot L., Frédérich M. (2010). In vitro and in vivo antimalarial and cytotoxic activity of five plants used in congolese traditional medicine. J. Ethnopharmacol..

[27] Odira H.O., Mitema S.O., Mapenay I.M., Moriasi G.A. (2022). Anti-inflammatory, analgesic, and cytotoxic effects of the phytexponent: A polyherbal formulation. J. Evid.-Based Integr. Med..

[28] Ngemenya M.N., Djeukem G.G.R., Nyongbela K.D., Bate P.N.N., Babiaka S.B., Monya E., Kanso R.K. (2019). Microbial, phytochemical, toxicity analyses and antibacterial activity against multidrug resistant bacteria of some traditional remedies sold in Buea Southwest Cameroon. BMC Complement. Altern. Med..

[29] Indrayanto G., Putra G.S., Suhud F., Al-Majed A.A. (2021). Chapter six-validation of in-vitro bioassay methods: Application in herbal drug research. Profiles of Drug Substances, Excipients and Related Methodology.

[30] Arya A., Kojom Foko L.P., Chaudhry S., Sharma A., Singh V. (2021). Artemisinin-based combination therapy (ACT) and drug resistance molecular markers: A systematic review of clinical studies from two malaria endemic regions-India and sub-Saharan Africa. Int. J. Parasitol. Drugs Drug Resist..

[31] Sarkar B., Chakraborty S., Pal C. (2021). Phytomedicine Against Infectious Diseases.

[32] Chemat F., Abert Vian M., Ravi H.K., Khadhraoui B., Hilali S., Perino S., Tixier A.-S.F. (2019). Review of alternative solvents for green extraction of food and natural products: Panorama, principles, applications and prospects. Molecules.

[33] Moore R.K., Smaglick J., Leitch E.A.-R.M., Mann D. The effect of polarity of extractives on the durability of wood. Proceedings of the 18th ISWFPC (International Symposium on Wood, Fiber, and Pulping).

[34] Abubakar A.R., Haque M. (2020). Preparation of medicinal plants: Basic extraction and fractionation procedures for experimental purposes. J. Pharm. Bioallied Sci..

[35] Mojarrab M., Naderi R., Heshmati Afshar F. (2015). Screening of different extracts from artemisia species for their potential antimalarial activity. Iran. J. Pharm. Res..

[36] Thiengsusuk A., Chaijaroenkul W., Na-Bangchang K. (2013). Antimalarial activities of medicinal plants and herbal formulations used in Thai traditional medicine. Parasitol. Res..

[37] Pinmai K., Hiriote W., Soonthornchareonnon N., Jongsakul K., Sireeratawong S., Tor-Udom S. (2010). In vitro and in vivo antiplasmodial activity and cytotoxicity of water extracts of *Phyllanthus emblica*, *Terminalia chebula*, and *Terminalia bellerica*. J. Med. Assoc. Thai..

[38] Aye M.M., Aung H.T., Sein M.M., Armijos C. (2019). A review on the phytochemistry, medicinal properties and pharmacological activities of 15 selected myanmar medicinal plants. Molecules.

[39] Agidew M.G. (2022). Phytochemical analysis of some selected traditional medicinal plants in Ethiopia. Bull. Natl. Res. Cent..

[40] Rudrapal M., Chetia D.D. (2016). Plant flavonoids as potential source of future antimalarial leads. Syst. Rev. Pharm..

[41] Yang W., Chen X., Li Y., Guo S., Wang Z., Yu X. (2020). Advances in pharmacological activities of Terpenoids. Nat. Prod. Commun..

[42] Jahangeer M., Fatima R., Ashiq M., Basharat A., Qamar S.A., Bilal M., Iqbal H.M.N. (2021). Therapeutic and biomedical potentialities of terpenoids—A review. J. Pure Appl. Microbiol..

[43] Murugan K., Panneerselvam C., Subramaniam J., Paulpandi M., Rajaganesh R., Vasanthakumaran M., Madhavan J., Shafi S.S., Roni M., Portilla-Pulido J.S. (2022). Synthesis of new series of quinoline derivatives with insecticidal effects on larval vectors of malaria and dengue diseases. Sci. Rep..

[44] Uzor P.F. (2020). Alkaloids from plants with antimalarial activity: A review of recent studies. Evid.-Based Complement. Altern. Med..

[45] Maugeri A., Lombardo G.E., Cirmi S., Süntar I., Barreca D., Laganà G., Navarra M. (2022). Pharmacology and toxicology of tannins. Arch. Toxicol..

[46] Lutgen P. (2018). Tannins in Artemisia: The hidden treasure of prophylaxis. Pharm. Pharmacol. Int..

[47] Mikhail N., Adewuyi A., Abdulsalam T., Ashafa T. (2021). Antimalarial activity and biochemical effects of saponin-rich extract of *Dianthus basuticus* Burtt Davy in *Plasmodium berghei*-infected mice. Adv. Tradit. Med..

[48] Hu X.L., Gao C., Xu Z., Liu M.L., Feng L.S., Zhang G.D. (2018). Recent development of coumarin derivatives as potential antiplasmodial and antimalarial agents. Curr. Top. Med. Chem..

[49] Shaikh J., Patil M. (2020). A review on phytotoxins and qualitative tests for their detection. Pharmacogn. Phytochem..

[50] Sung J.H. (2021). Multi-organ-on-a-chip for pharmacokinetics and toxicokinetic study of drugs. Expert Opin. Drug Metab. Toxicol..

[51] Mahavorasirikul W., Viyanant V., Chaijaroenkul W., Itharat A., Na-Bangchang K. (2010). Cytotoxic activity of Thai medicinal plants against human cholangiocarcinoma, laryngeal and hepatocarcinoma cells in vitro. BMC Complement. Altern. Med..

[52] Ruangnoo S., Itharat A., Sakpakdeejaroen I., Rattarom R., Tappayutpijam P., Pawa K.K. (2012). In vitro cytotoxic activity of Benjakul herbal preparation and its active compounds against human lung, cervical and liver cancer cells. J. Med. Assoc. Thai..

[53] Saraswati S., Kanaujia P., Sunder S. (2013). OP-03 α-Santalol demonstrates antitumor and antiantiangiogenic activities in models of hepatocellular carcinoma in vitro and in vivo. Dig. Liver Dis..

[54] Hwang B.Y., Su B.-N., Chai H., Mi Q., Kardono L.B.S., Afriastini J.J., Riswan S., Santarsiero B.D., Mesecar A.D., Wild R. (2004). Silvestrol and episilvestrol, potential anticancer rocaglate derivatives from *Aglaia silvestris*. J. Org. Chem..

[55] Chen X., Winstead A., Yu H., Peng J. (2021). Taccalonolides: A novel class of microtubule-stabilizing anticancer agents. Cancers.

[56] Gopalakrishnan A.M., Kumar N. (2015). Antimalarial action of artesunate involves DNA damage mediated by reactive oxygen species. Antimicrob. Agents Chemother..

[57] Yang S., Lian G. (2020). ROS and diseases: Role in metabolism and energy supply. Mol. Cell. Biochem..

[58] Cock I.E. (2015). The medicinal properties and phytochemistry of plants of the genus Terminalia (Combretaceae). Inflammopharmacology.

[59] Peña-Morán O.A., Villarreal M.L., Álvarez-Berber L., Meneses-Acosta A., Rodríguez-López V. (2016). Cytotoxicity, post-treatment recovery, and selectivity analysis of naturally occurring podophyllotoxins from *Bursera fagaroides* var. fagaroides on breast cancer cell lines. Molecules.

[60] Basir R., Rahiman S.F., Hasballah K., Chong W., Talib H., Yam M., Jabbarzare M., Tie T., Othman F., Moklas M. (2012). *Plasmodium berghei* ANKA infection in ICR mice as a model of cerebral malaria. Iran. J. Parasitol..

[61] Khasanah U., WidyaWaruyanti A., Hafid A.F., Tanjung M. (2017). Antiplasmodial activity of isolated polyphenols from *Alectryon serratus* leaves against 3D7 *Plasmodium falciparum*. Pharmacogn. Res..

[62] Soh P.N., Witkowski B., Olagnier D., Nicolau M.L., Garcia-Alvarez M.C., Berry A., Benoit-Vical F. (2009). In vitro and in vivo properties of ellagic acid in malaria treatment. Antimicrob. Agents Chemother..

[63] Belapurkar P., Goyal P., Tiwari-Barua P. (2014). Immunomodulatory effects of triphala and its individual constituents: A review. Indian J. Pharm. Sci..

[64] Baliga M.S., Meera S., Mathai B., Rai M.P., Pawar V., Palatty P.L. (2012). Scientific validation of the ethnomedicinal properties of the Ayurvedic drug Triphala: A review. Chin. J. Integr. Med..

[65] Lazic S.E., Semenova E., Williams D.P. (2020). Determining organ weight toxicity with Bayesian causal models: Improving on the analysis of relative organ weights. Sci. Rep..

